# Author Correction: Cell-mediated exon skipping normalizes dystrophin expression and muscle function in a new mouse model of Duchenne Muscular Dystrophy

**DOI:** 10.1038/s44321-024-00134-x

**Published:** 2024-09-06

**Authors:** Francesco Galli, Laricia Bragg, Maira Rossi, Daisy Proietti, Laura Perani, Marco Bacigaluppi, Rossana Tonlorenzi, Tendai Sibanda, Miriam Caffarini, Avraneel Talapatra, Sabrina Santoleri, Mirella Meregalli, Beatriz Bano-Otalora, Anne Bigot, Irene Bozzoni, Chiara Bonini, Vincent Mouly, Yvan Torrente, Giulio Cossu

**Affiliations:** 1 Division of Cell Matrix Biology & Regenerative Medicine, Faculty of Biology, Medicine and Health, University of Manchester, Manchester, UK; 2 Institute of Experimental Neurology, Division of Neurosciences, IRCCS San Raffaele Scientific Institute, Milan, Italy; 3 Department of Pathophysiology and Transplantation, Università degli Studi di Milano, Fondazione IRCCS Ca’ Granda Ospedale Maggiore Policlinico, Centro Dino Ferrari, 20122 Milan, Italy; 4 Division of Neuroscience and Experimental Psychology, School of Biological Sciences, Faculty of Biology, Medicine and Health, University of Manchester, Manchester, UK; 5 Institut de Myologie, Université Pierre et Marie Curie, Paris 6 UM76, Univ. Paris 6/U974, UMR7215, CNRS, Pitié-Salpétrière-INSERM, UMRS 974 Paris, France; 6 Department of Biology and Biotechnology Charles Darwin, Sapienza University of Rome, 00161 Rome, Italy; 7 Center for Life Nano- & Neuro-Science@Sapienza of Istituto Italiano di Tecnologia (IIT), 00161 Rome, Italy; 8 Experimental Hematology Unit, Vita-Salute San Raffaele University, Milan, Italy; 9 IRCCS Ospedale San Raffaele Scientific Institute, 20133 Milan, Italy; 10 Experimental and Clinical Research Center. Charité Medical Faculty and Max Delbrück Center 13125 Berlin, Berlin, Germany

## Abstract

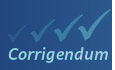

**Correction to:** *EMBO Molecular Medicine* (2024) 16:927–944. 10.1038/s44321-024-00031-3 | Published online 4 March 2024

**An author’s name is corrected**

**Author affiliation number** ^**2**^ **is corrected**

The author name Marco Bagicaluppi is corrected to:

**Marco Bacigaluppi**.

Author affiliation number ^2^ is corrected from: ^2^Institue of Experimental Neurology, Division of Neurosciences. Ospedale San Raffaele Milan, Italy

To: (changes in bold)

^**2**^ **Institute of Experimental Neurology, Division of Neurosciences, IRCCS San Raffaele Scientific Institute, Milan, Italy**

