# Supplementary figures and images for: Cell-mediated exon skipping normalizes dystrophin expression and muscle function in a new mouse model of Duchenne Muscular Dystrophy
Source: EMBO Mol Med. 2024 Mar 4;16(4):19. doi: 10.1038/s44321-024-00031-3 (PMC11018779; doi:10.1038/s44321-024-00031-3)

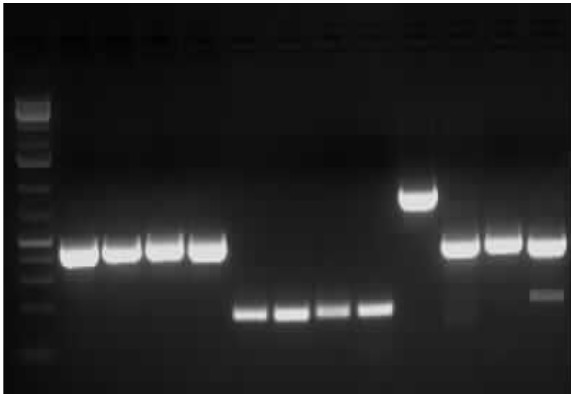

Supplement: Supplementary file 1 — Source Data Fig. 1 [file 44321_2024_31_MOESM1_ESM.zip › Figure 1/1A/PCR.jpg]

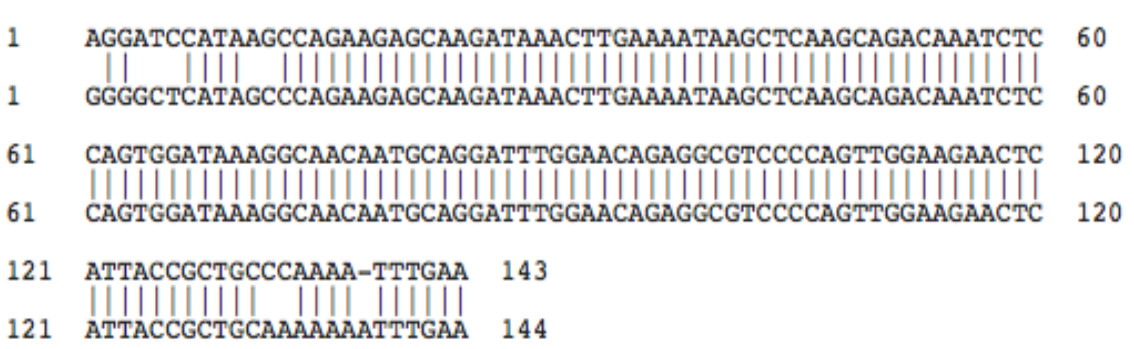

Supplement: Supplementary file 1 — Source Data Fig. 1 [file 44321_2024_31_MOESM1_ESM.zip › Figure 1/1B/Sequencing.jpg]

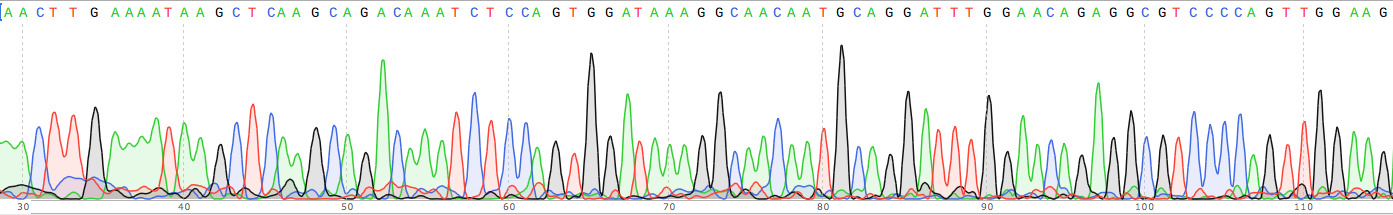

Supplement: Supplementary file 1 — Source Data Fig. 1 [file 44321_2024_31_MOESM1_ESM.zip › Figure 1/1B/Sequencing Spectrum.png]

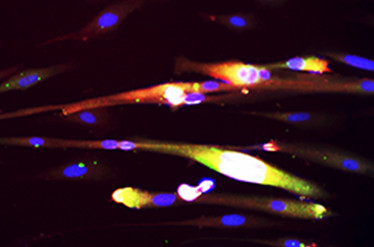

Supplement: Supplementary file 1 — Source Data Fig. 1 [file 44321_2024_31_MOESM1_ESM.zip › Figure 1/1D/51-DMD 1:30 Dapi:GFP:Dys Magnification.jpg]

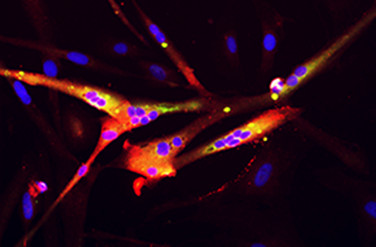

Supplement: Supplementary file 1 — Source Data Fig. 1 [file 44321_2024_31_MOESM1_ESM.zip › Figure 1/1D/51-DMD 1:10 Dapi:GFP:Dys Magnification.jpg]

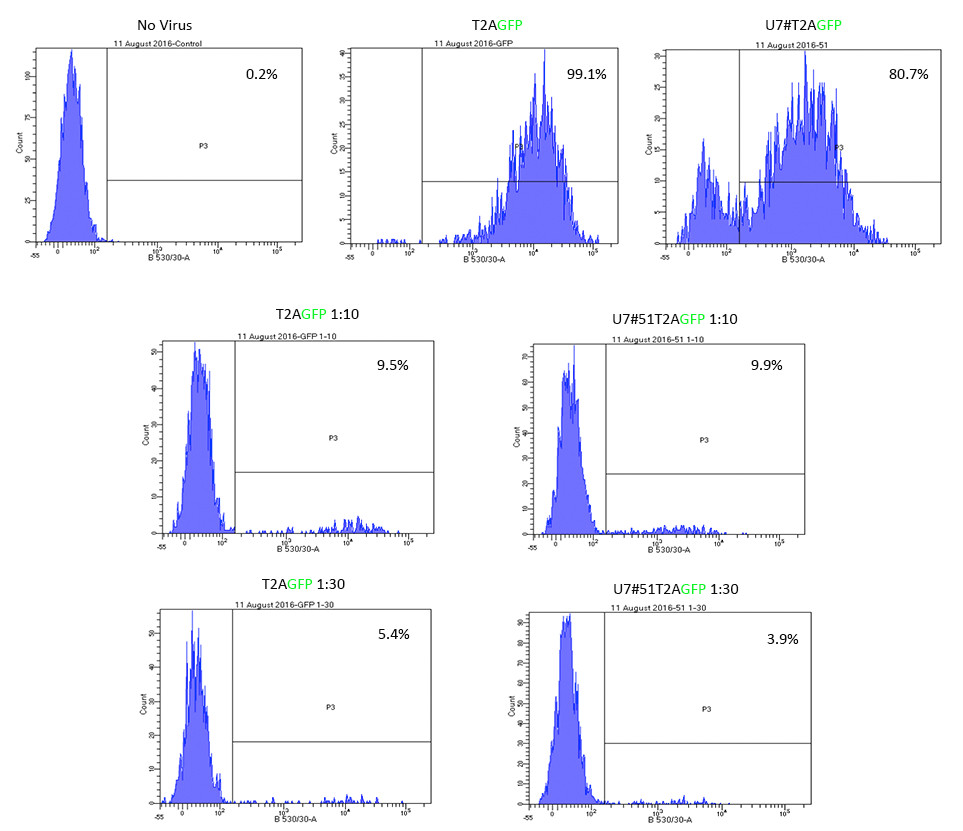

Supplement: Supplementary file 1 — Source Data Fig. 1 [file 44321_2024_31_MOESM1_ESM.zip › Figure 1/1C/FACs Analysis GFP Cells .jpg]

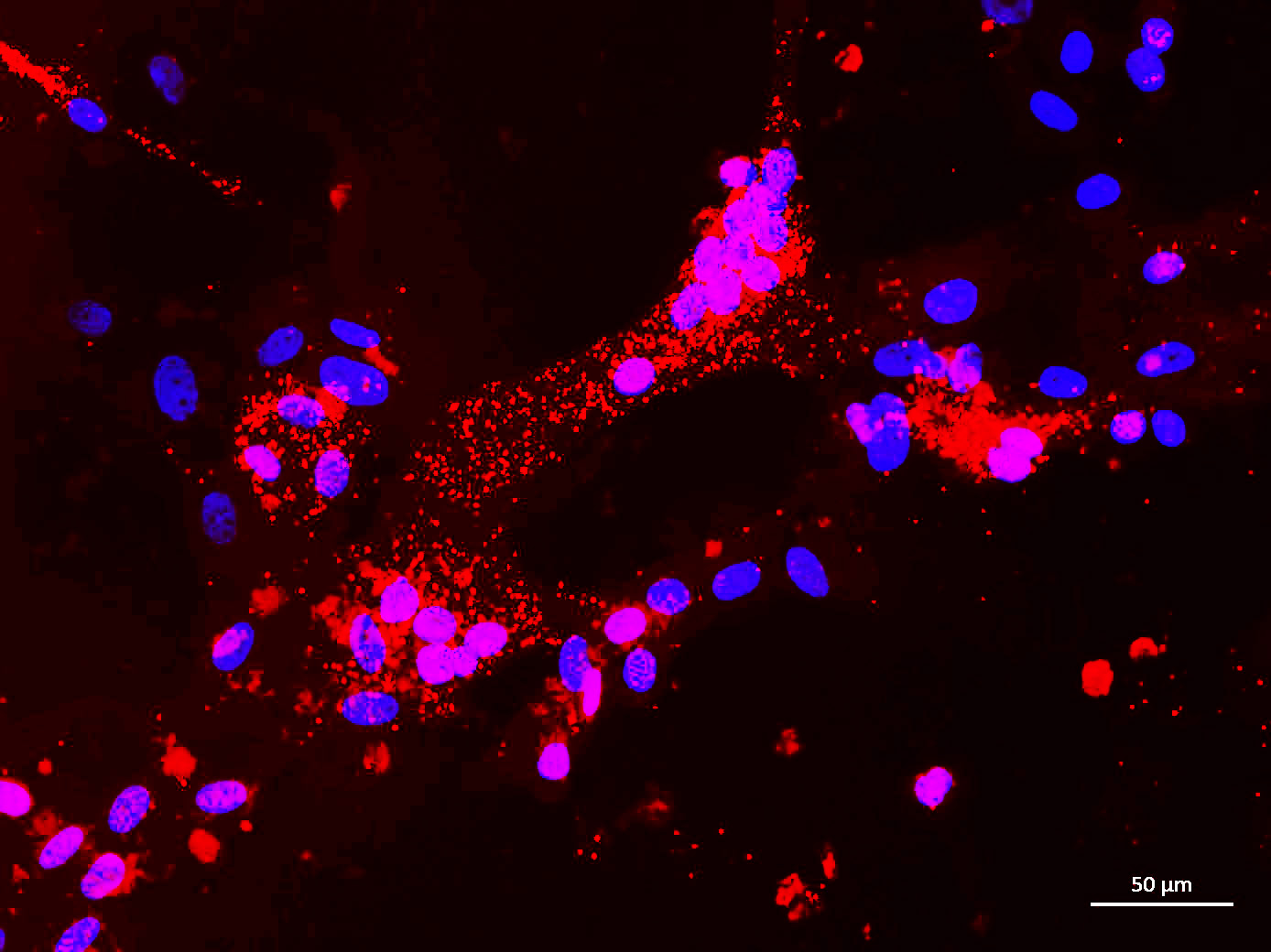

Supplement: Supplementary file 2 — Source Data Fig. 2 [file 44321_2024_31_MOESM2_ESM.zip › Figure 2/2A/U7 in situ.jpg]

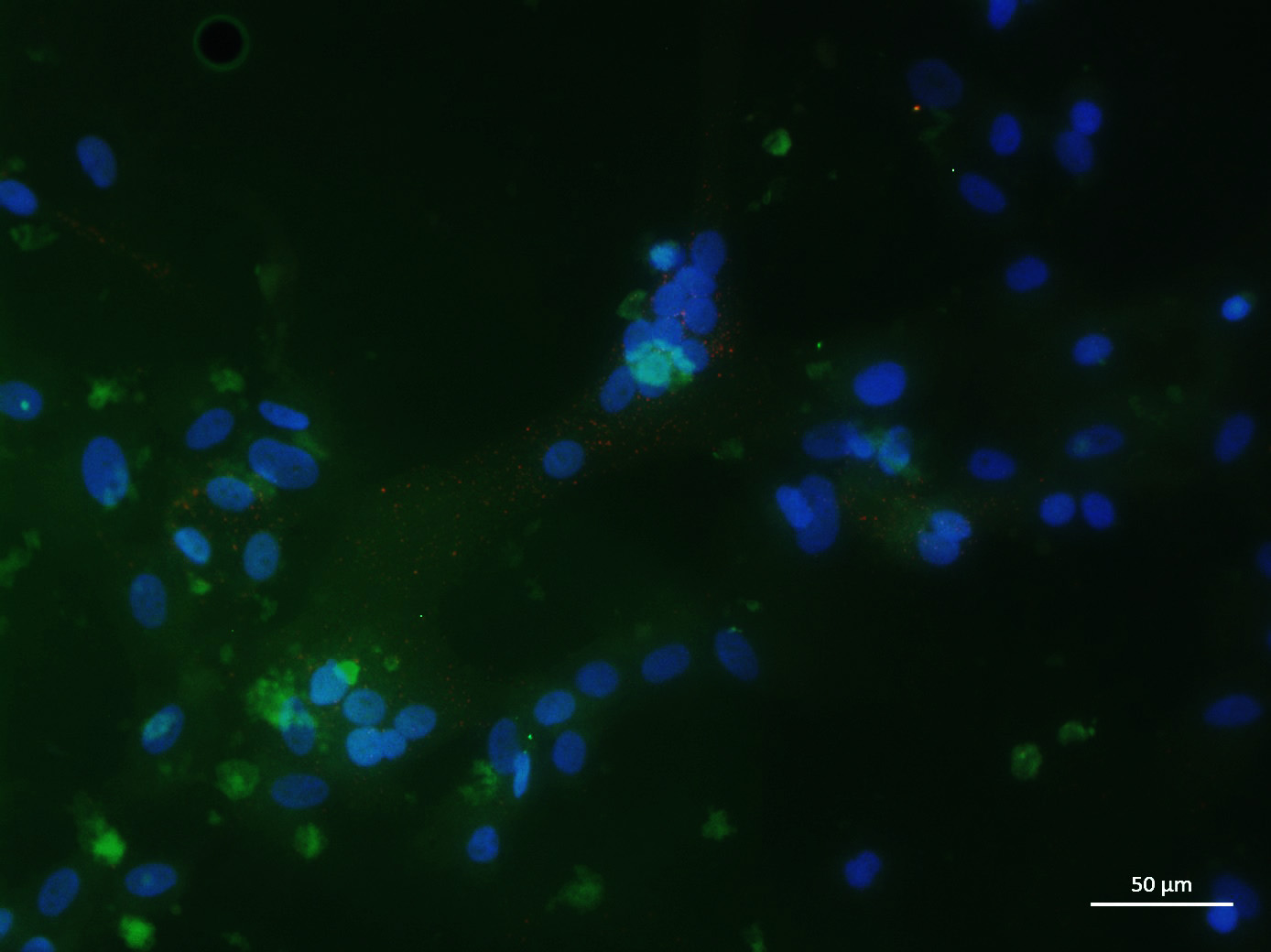

Supplement: Supplementary file 2 — Source Data Fig. 2 [file 44321_2024_31_MOESM2_ESM.zip › Figure 2/2A/GFP in situ.jpg]

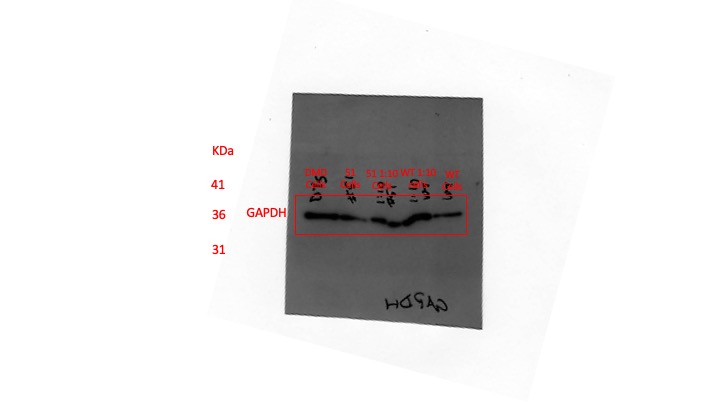

Supplement: Supplementary file 2 — Source Data Fig. 2 [file 44321_2024_31_MOESM2_ESM.zip › Figure 2/2C/GAPDH WB.jpg]

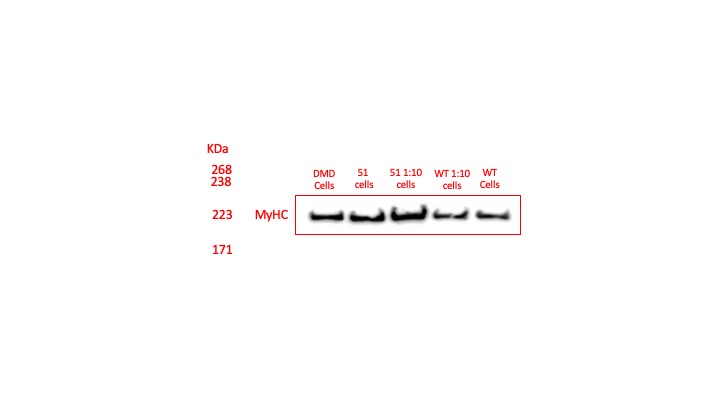

Supplement: Supplementary file 2 — Source Data Fig. 2 [file 44321_2024_31_MOESM2_ESM.zip › Figure 2/2C/MyHC WB.jpg]

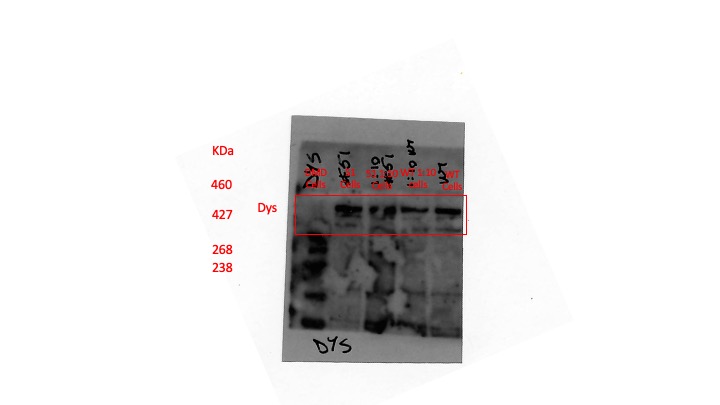

Supplement: Supplementary file 2 — Source Data Fig. 2 [file 44321_2024_31_MOESM2_ESM.zip › Figure 2/2C/Dys WB.jpg]

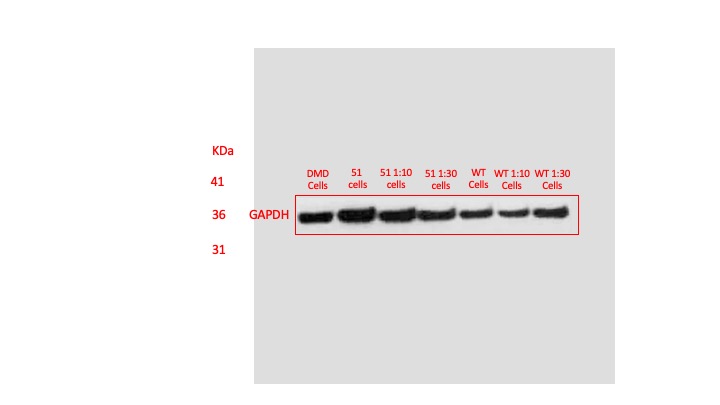

Supplement: Supplementary file 2 — Source Data Fig. 2 [file 44321_2024_31_MOESM2_ESM.zip › Figure 2/2D/GAPDH WB.jpg]

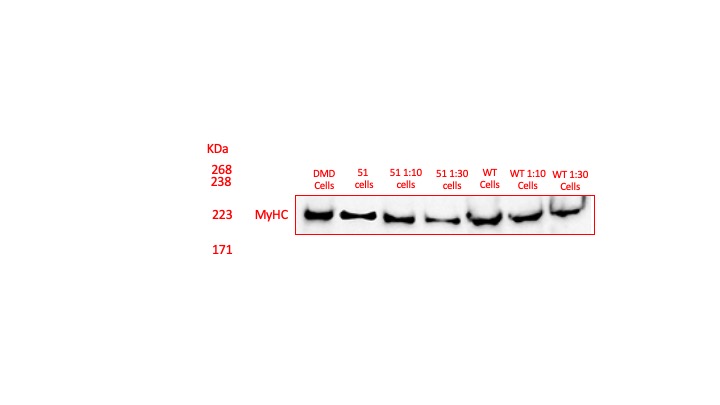

Supplement: Supplementary file 2 — Source Data Fig. 2 [file 44321_2024_31_MOESM2_ESM.zip › Figure 2/2D/MyHC WB.jpg]

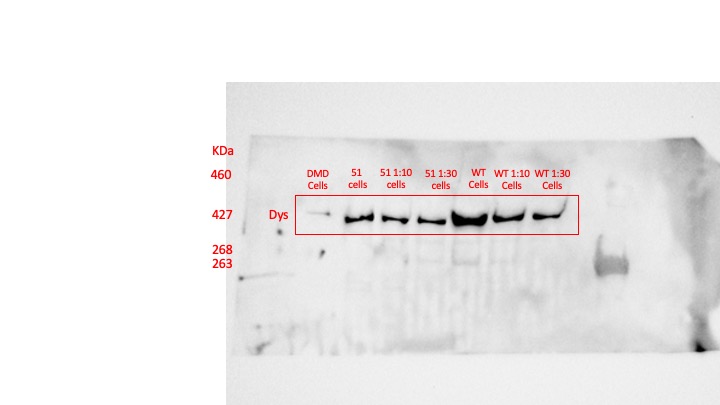

Supplement: Supplementary file 2 — Source Data Fig. 2 [file 44321_2024_31_MOESM2_ESM.zip › Figure 2/2D/Dys WB.jpg]

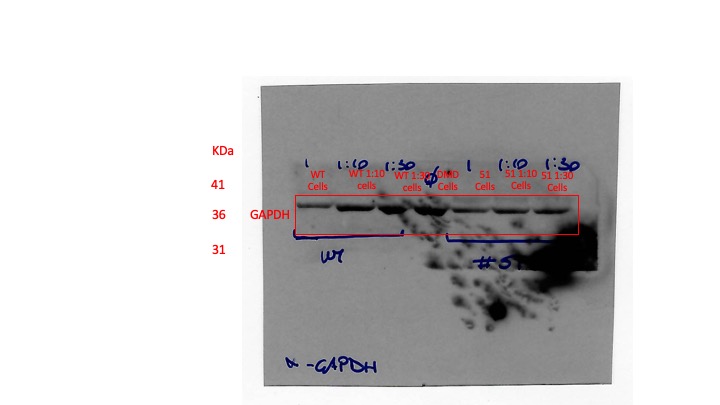

Supplement: Supplementary file 2 — Source Data Fig. 2 [file 44321_2024_31_MOESM2_ESM.zip › Figure 2/2B/GAPDH WB.jpg]

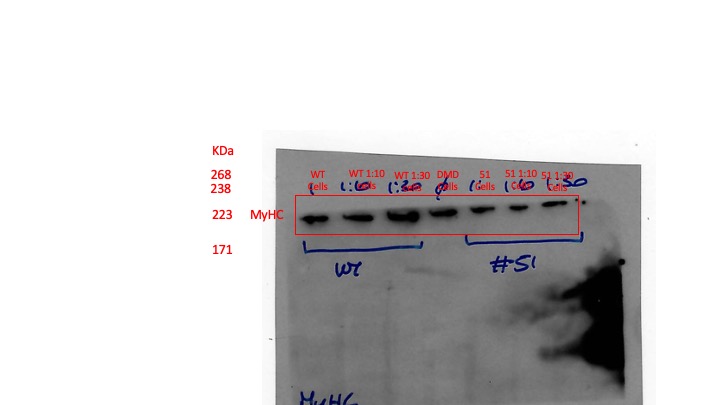

Supplement: Supplementary file 2 — Source Data Fig. 2 [file 44321_2024_31_MOESM2_ESM.zip › Figure 2/2B/MyHC WB.jpg]

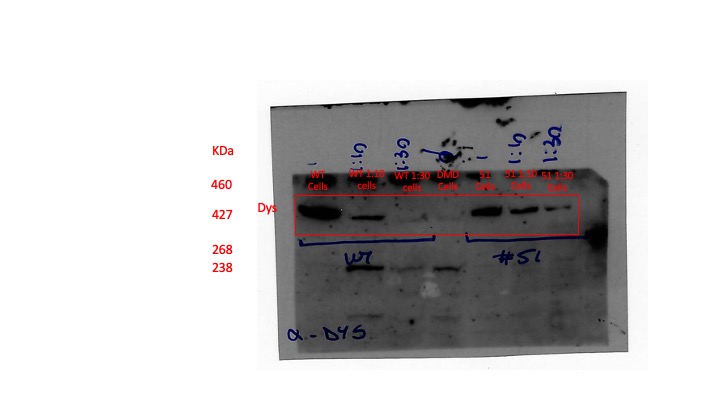

Supplement: Supplementary file 2 — Source Data Fig. 2 [file 44321_2024_31_MOESM2_ESM.zip › Figure 2/2B/Dys WB.jpg]

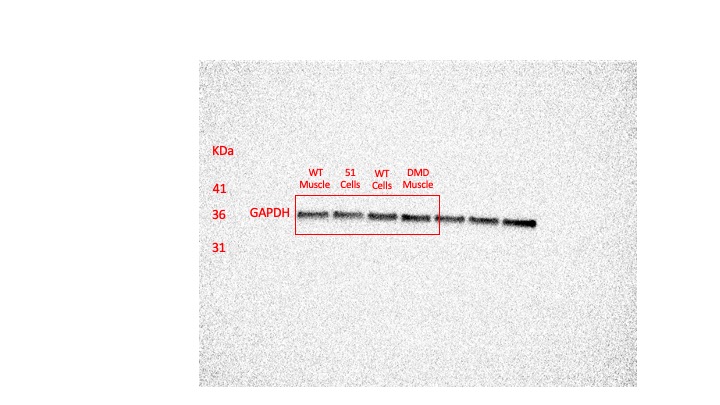

Supplement: Supplementary file 3 — Source Data Fig. 3 [file 44321_2024_31_MOESM3_ESM.zip › Figure 3/3B/GAPDH WB.jpg]

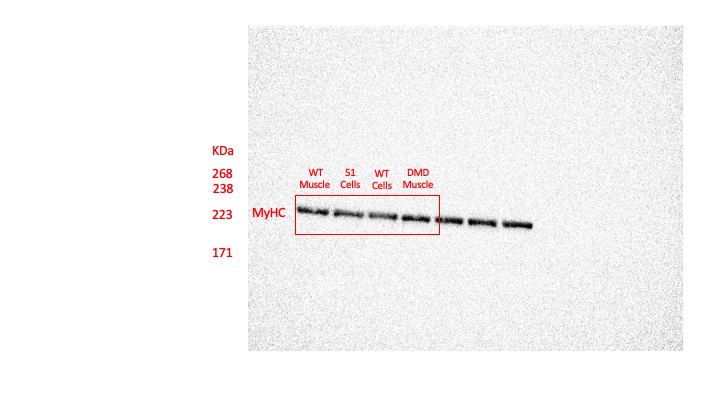

Supplement: Supplementary file 3 — Source Data Fig. 3 [file 44321_2024_31_MOESM3_ESM.zip › Figure 3/3B/MyHC WB.jpg]

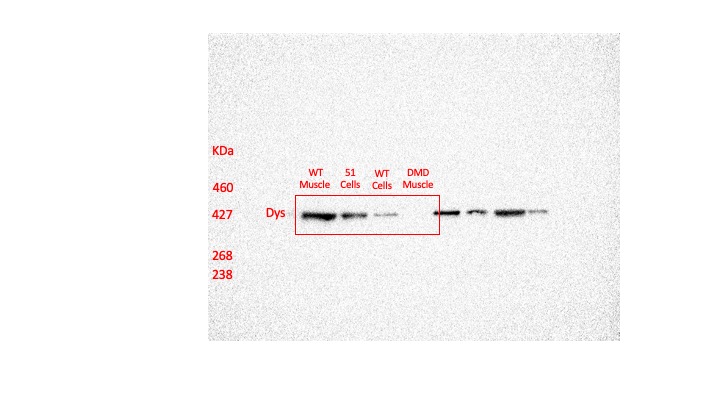

Supplement: Supplementary file 3 — Source Data Fig. 3 [file 44321_2024_31_MOESM3_ESM.zip › Figure 3/3B/Dys WB.jpg]

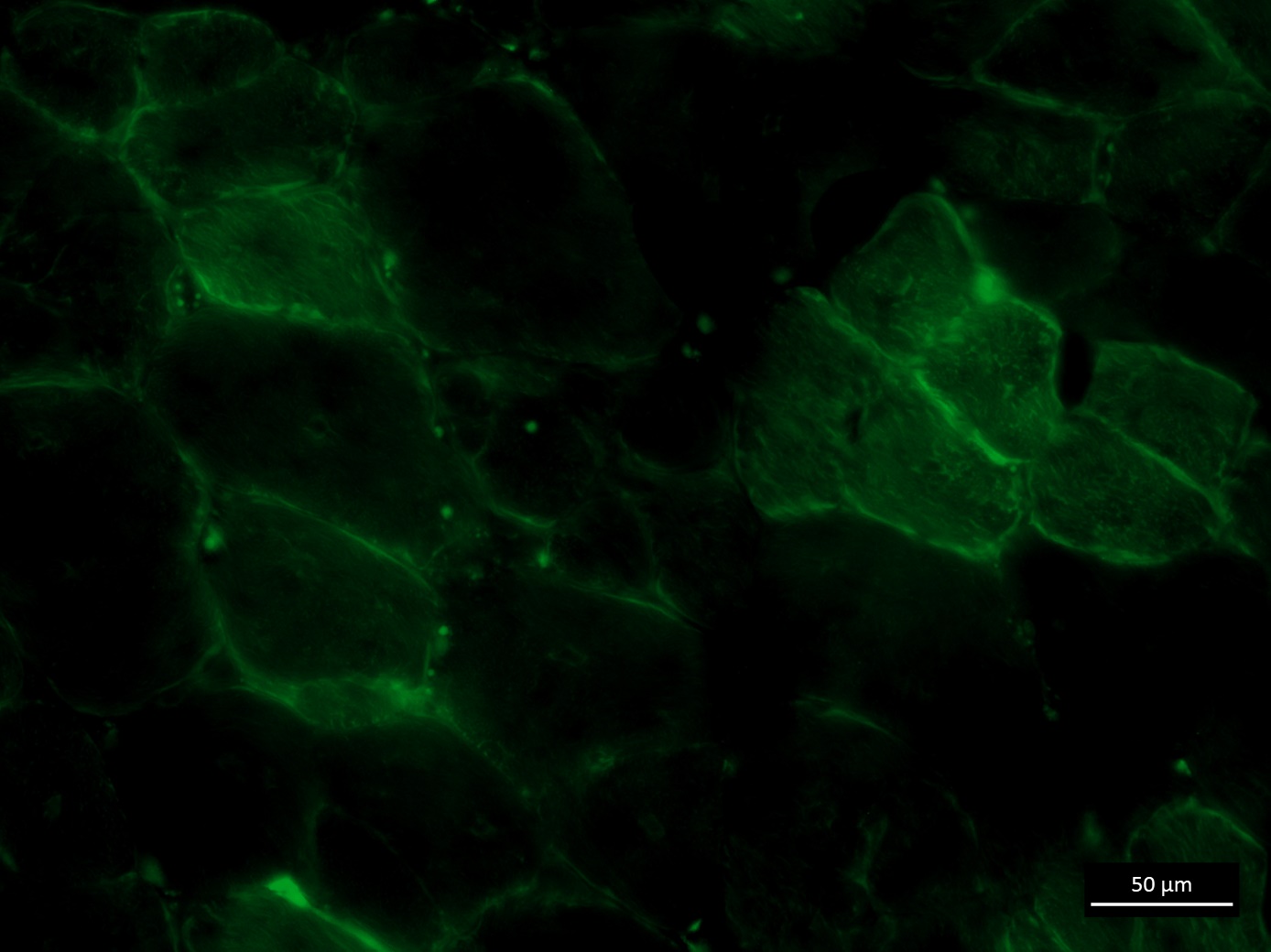

Supplement: Supplementary file 3 — Source Data Fig. 3 [file 44321_2024_31_MOESM3_ESM.zip › Figure 3/3C/WT Dys.jpg]

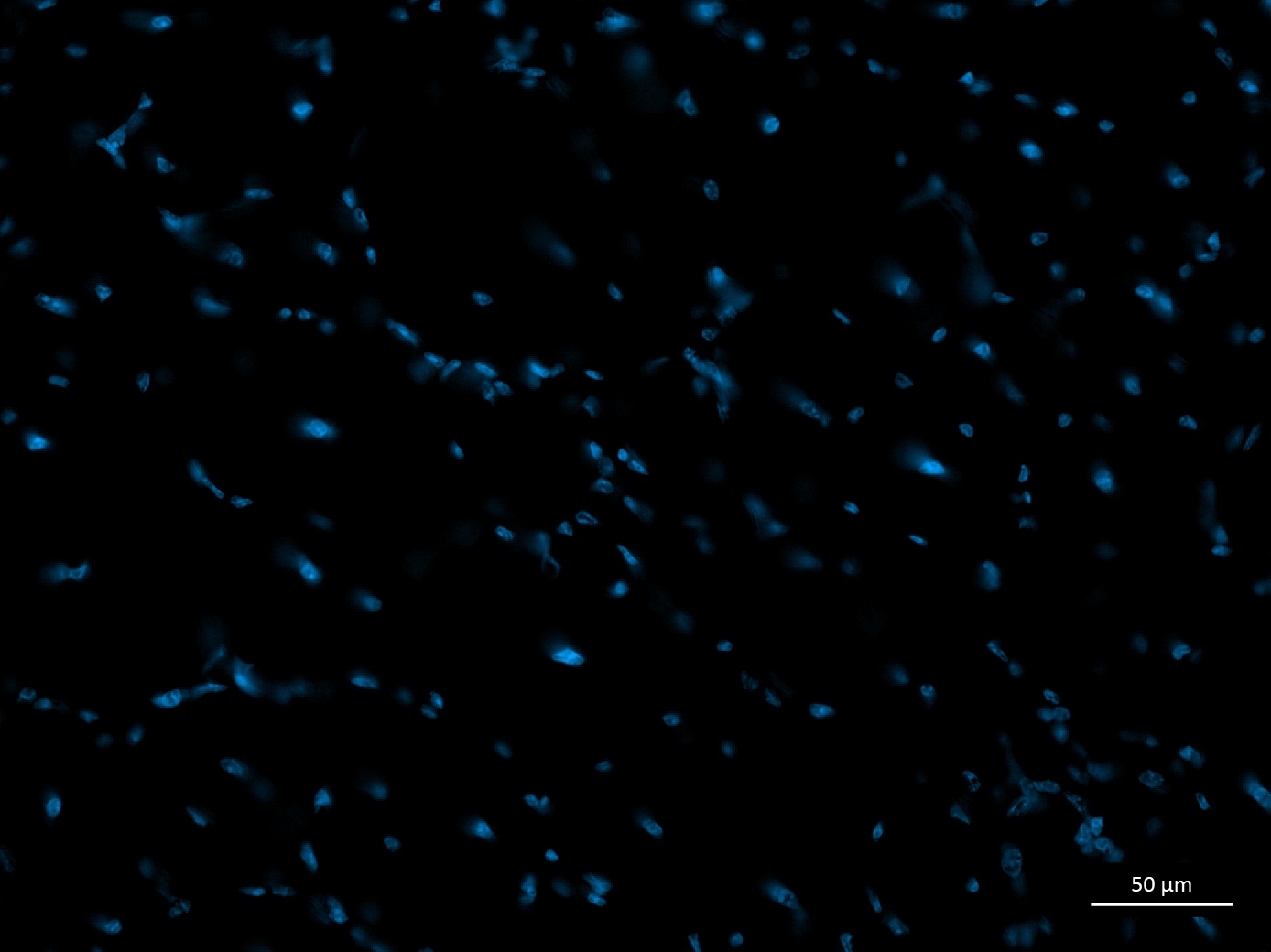

Supplement: Supplementary file 3 — Source Data Fig. 3 [file 44321_2024_31_MOESM3_ESM.zip › Figure 3/3C/WT Dapi.jpg]

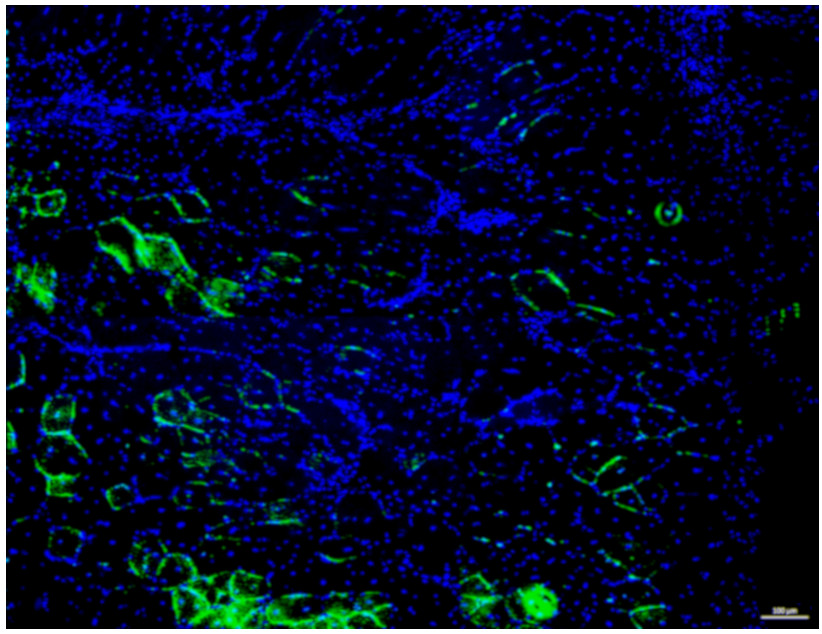

Supplement: Supplementary file 3 — Source Data Fig. 3 [file 44321_2024_31_MOESM3_ESM.zip › Figure 3/3C/WT DAPI:Dys Tile.jpg]

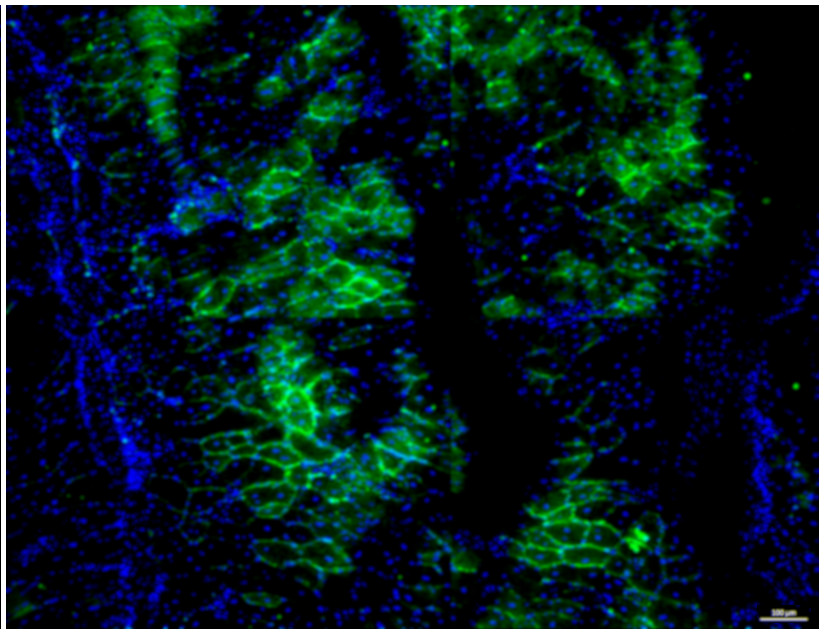

Supplement: Supplementary file 3 — Source Data Fig. 3 [file 44321_2024_31_MOESM3_ESM.zip › Figure 3/3C/51 DAPI:Dys Tile .jpg]

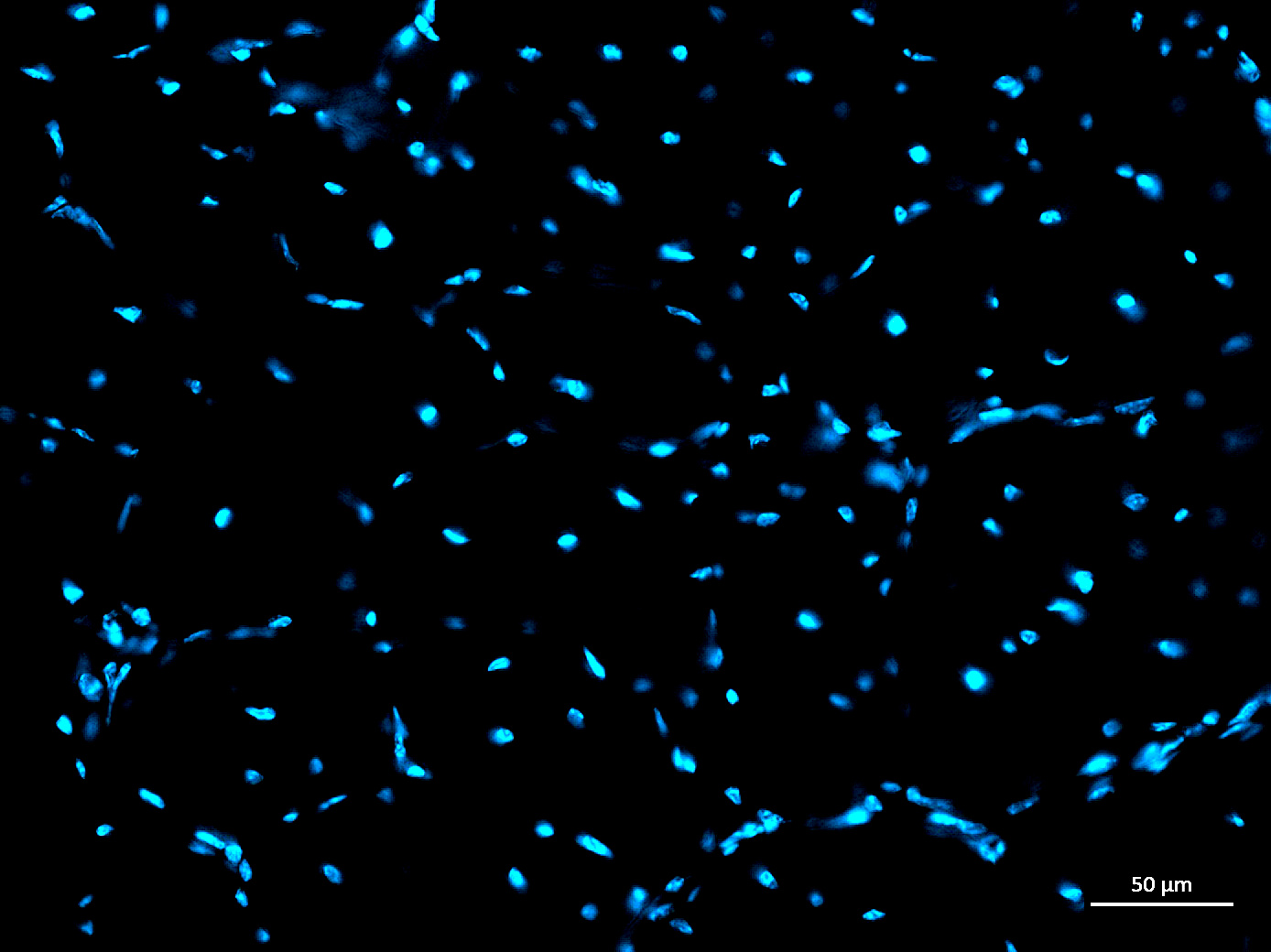

Supplement: Supplementary file 3 — Source Data Fig. 3 [file 44321_2024_31_MOESM3_ESM.zip › Figure 3/3C/51 Dapi.jpg]

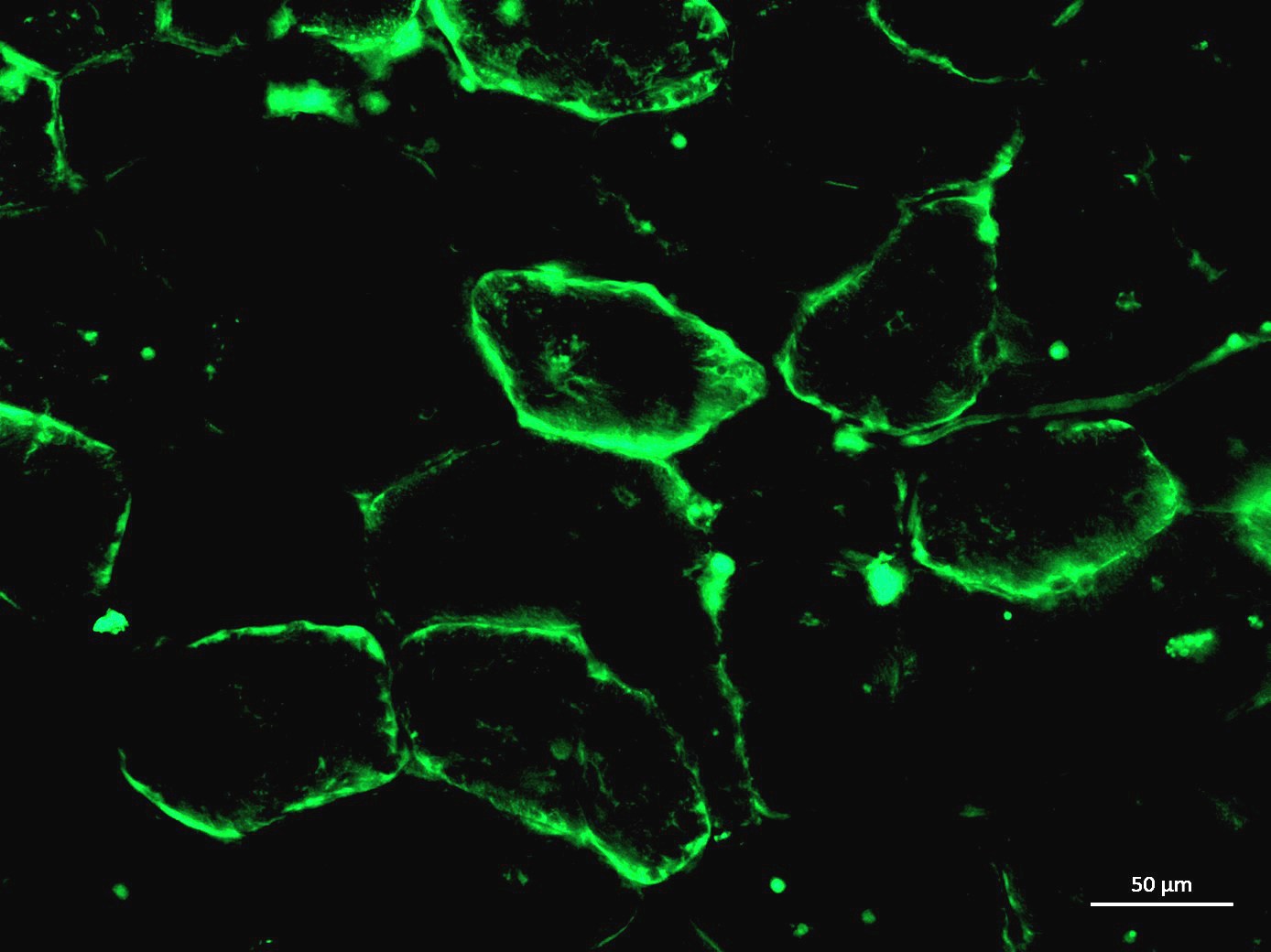

Supplement: Supplementary file 3 — Source Data Fig. 3 [file 44321_2024_31_MOESM3_ESM.zip › Figure 3/3C/51 Dys.jpg]

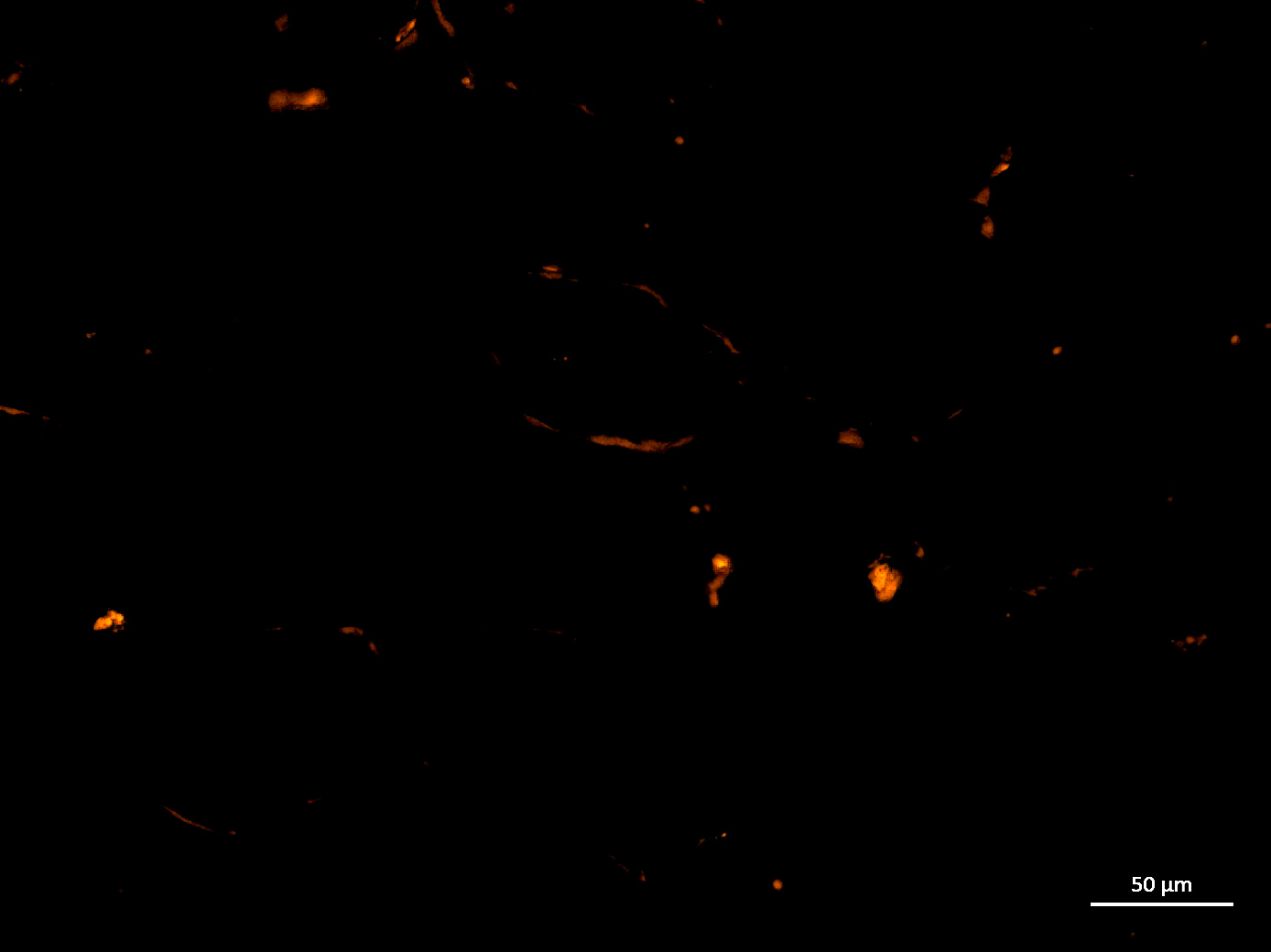

Supplement: Supplementary file 3 — Source Data Fig. 3 [file 44321_2024_31_MOESM3_ESM.zip › Figure 3/3C/51 Lam AC.jpg]

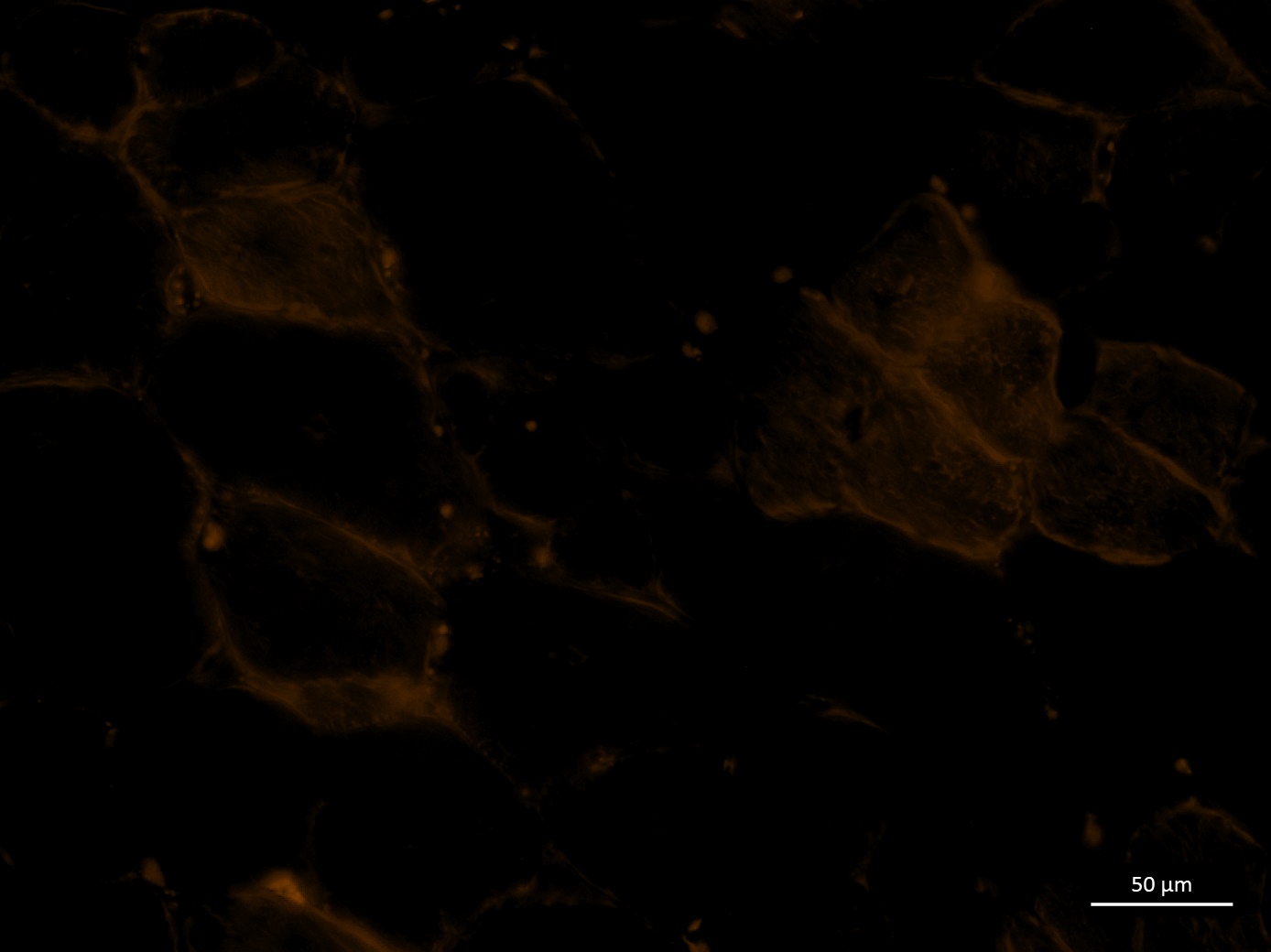

Supplement: Supplementary file 3 — Source Data Fig. 3 [file 44321_2024_31_MOESM3_ESM.zip › Figure 3/3C/WT Lam AC.jpg]

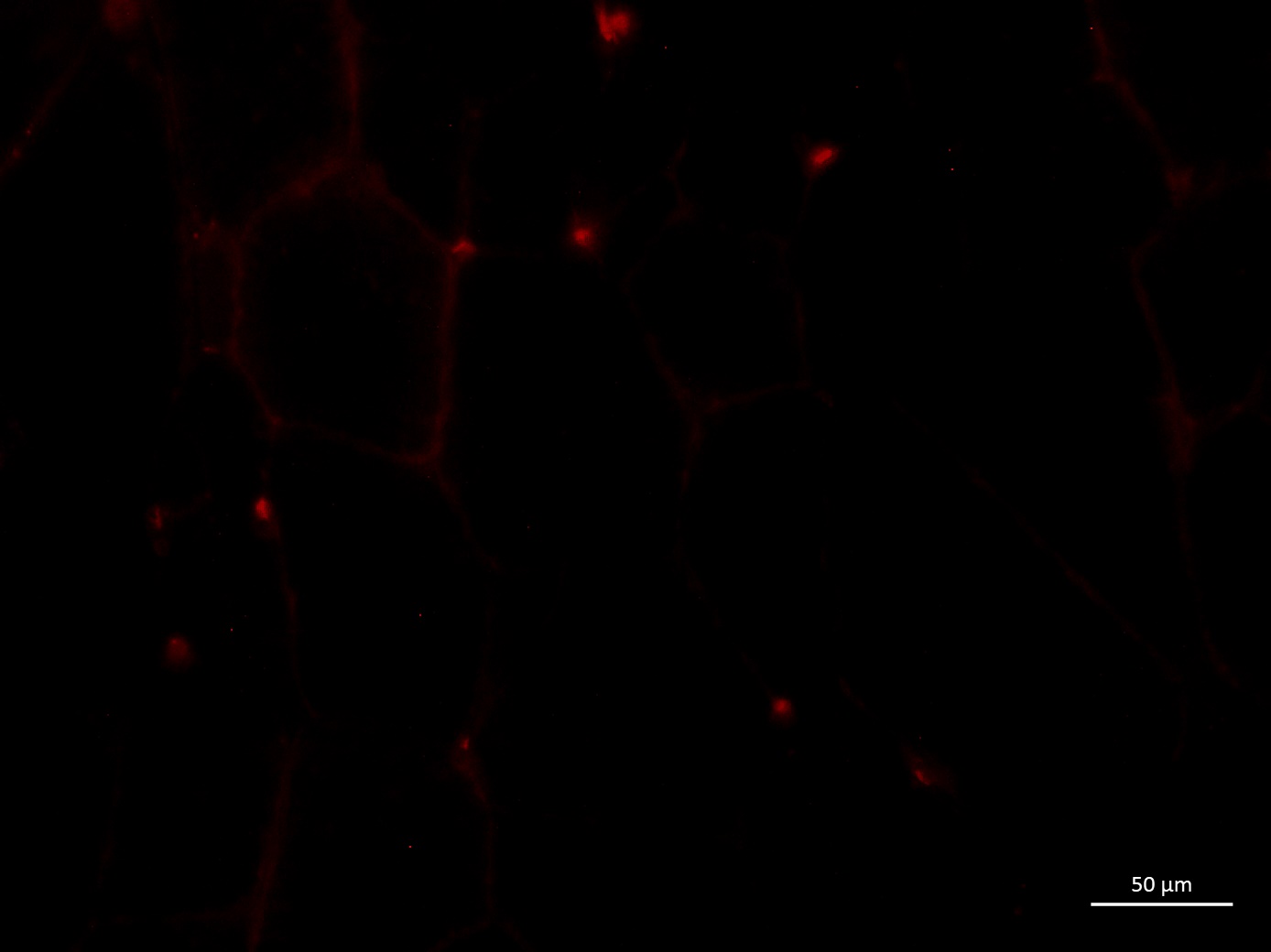

Supplement: Supplementary file 3 — Source Data Fig. 3 [file 44321_2024_31_MOESM3_ESM.zip › Figure 3/3D/WT nNOS.jpg]

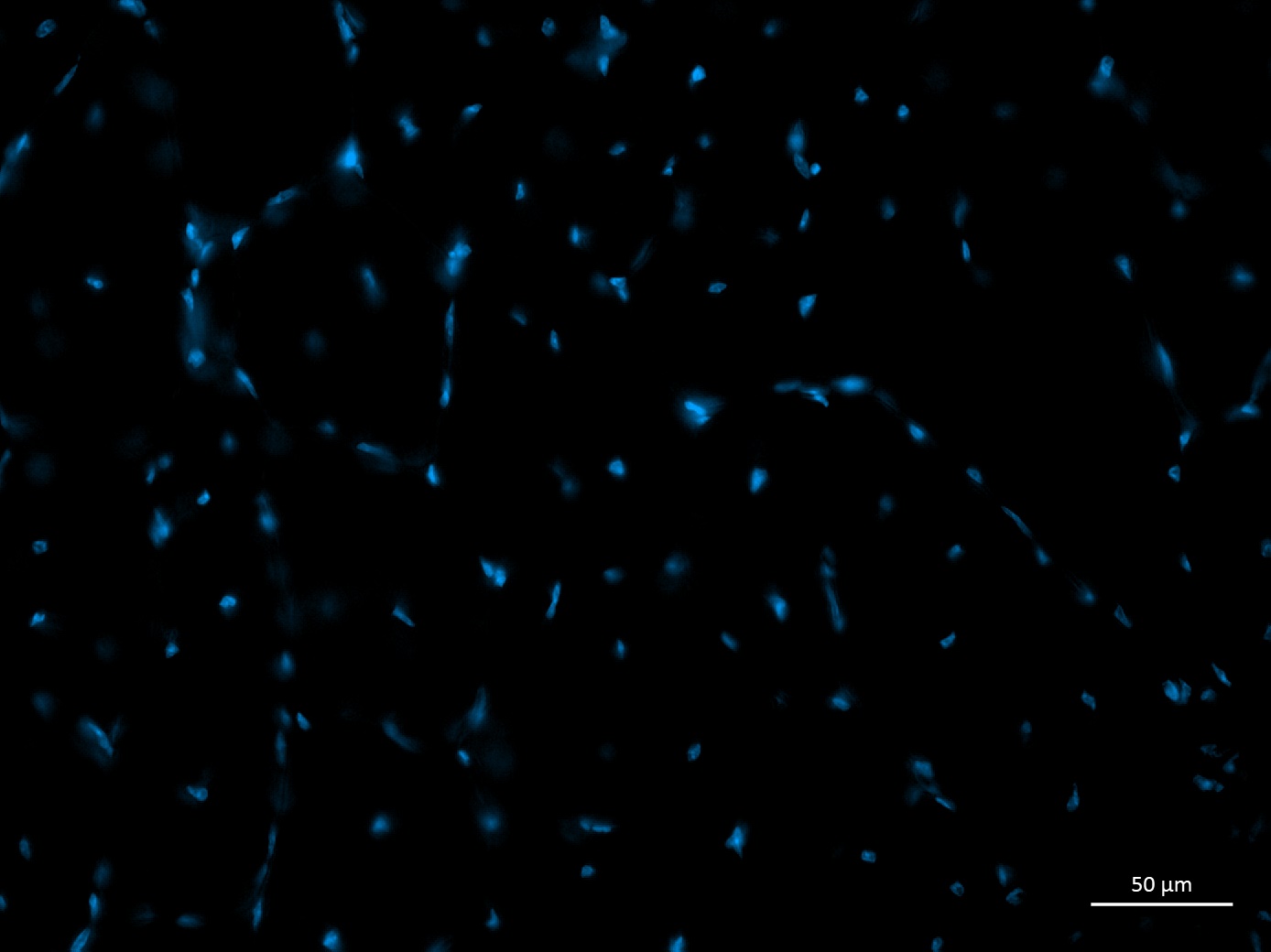

Supplement: Supplementary file 3 — Source Data Fig. 3 [file 44321_2024_31_MOESM3_ESM.zip › Figure 3/3D/WT Dapi.jpg]

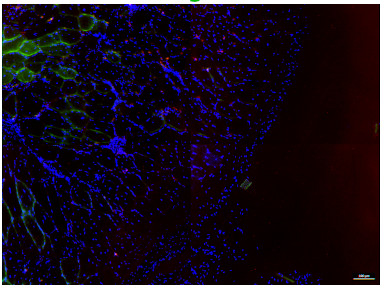

Supplement: Supplementary file 3 — Source Data Fig. 3 [file 44321_2024_31_MOESM3_ESM.zip › Figure 3/3D/WT Dapi:nNOS:AlphaSG Tile.jpg]

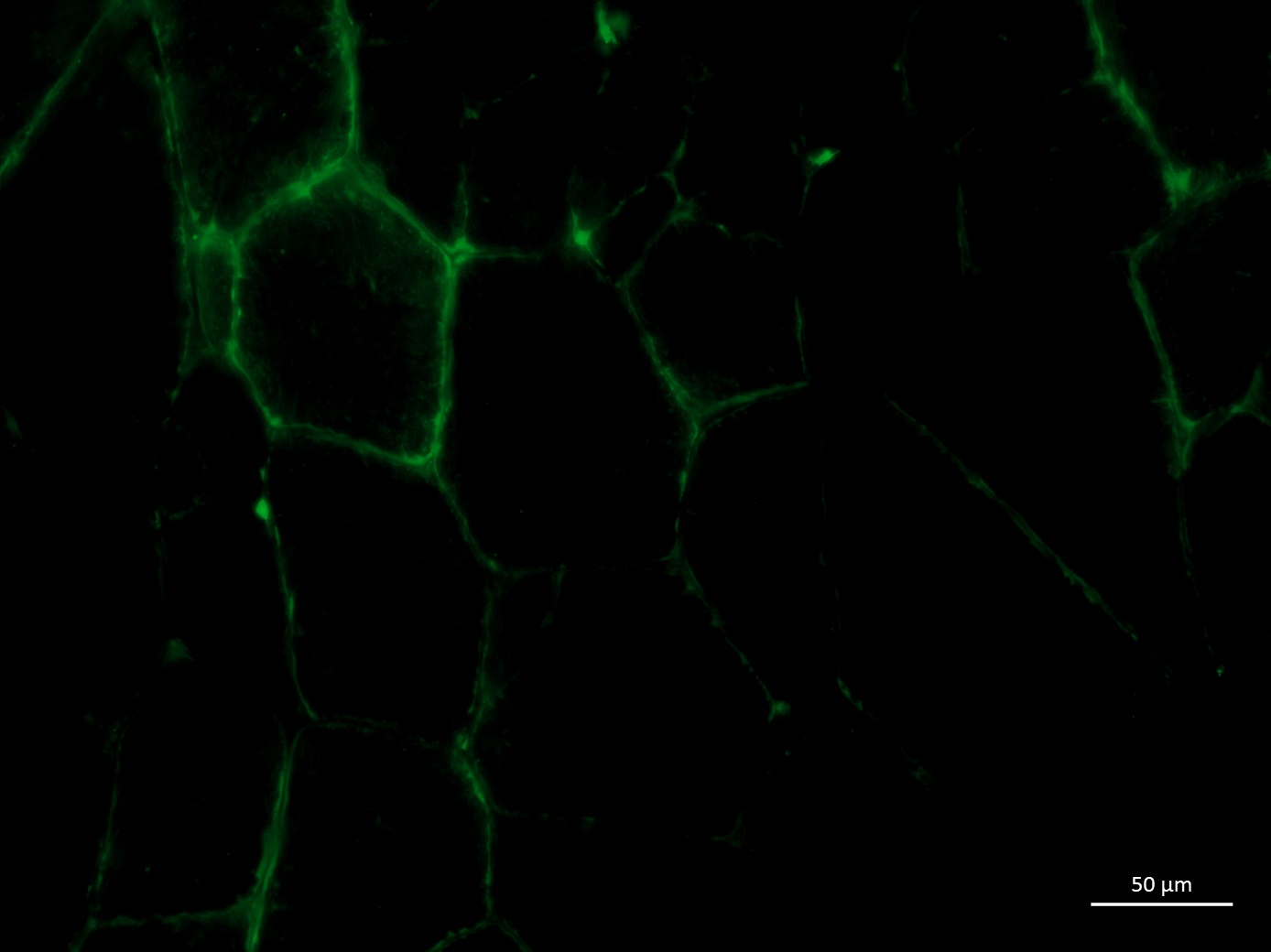

Supplement: Supplementary file 3 — Source Data Fig. 3 [file 44321_2024_31_MOESM3_ESM.zip › Figure 3/3D/WT AlphaSG.jpg]

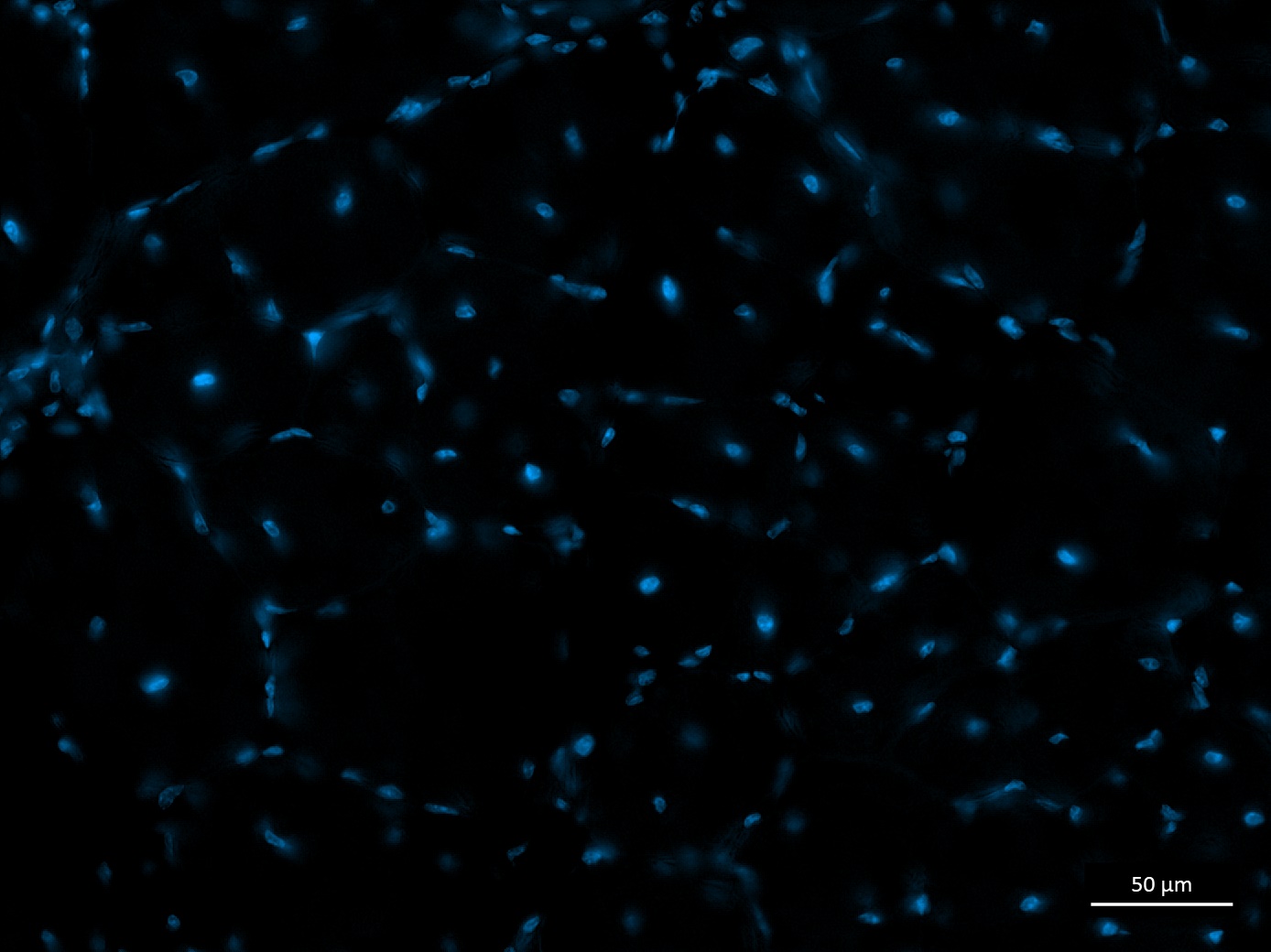

Supplement: Supplementary file 3 — Source Data Fig. 3 [file 44321_2024_31_MOESM3_ESM.zip › Figure 3/3D/51 Dapi.jpg]

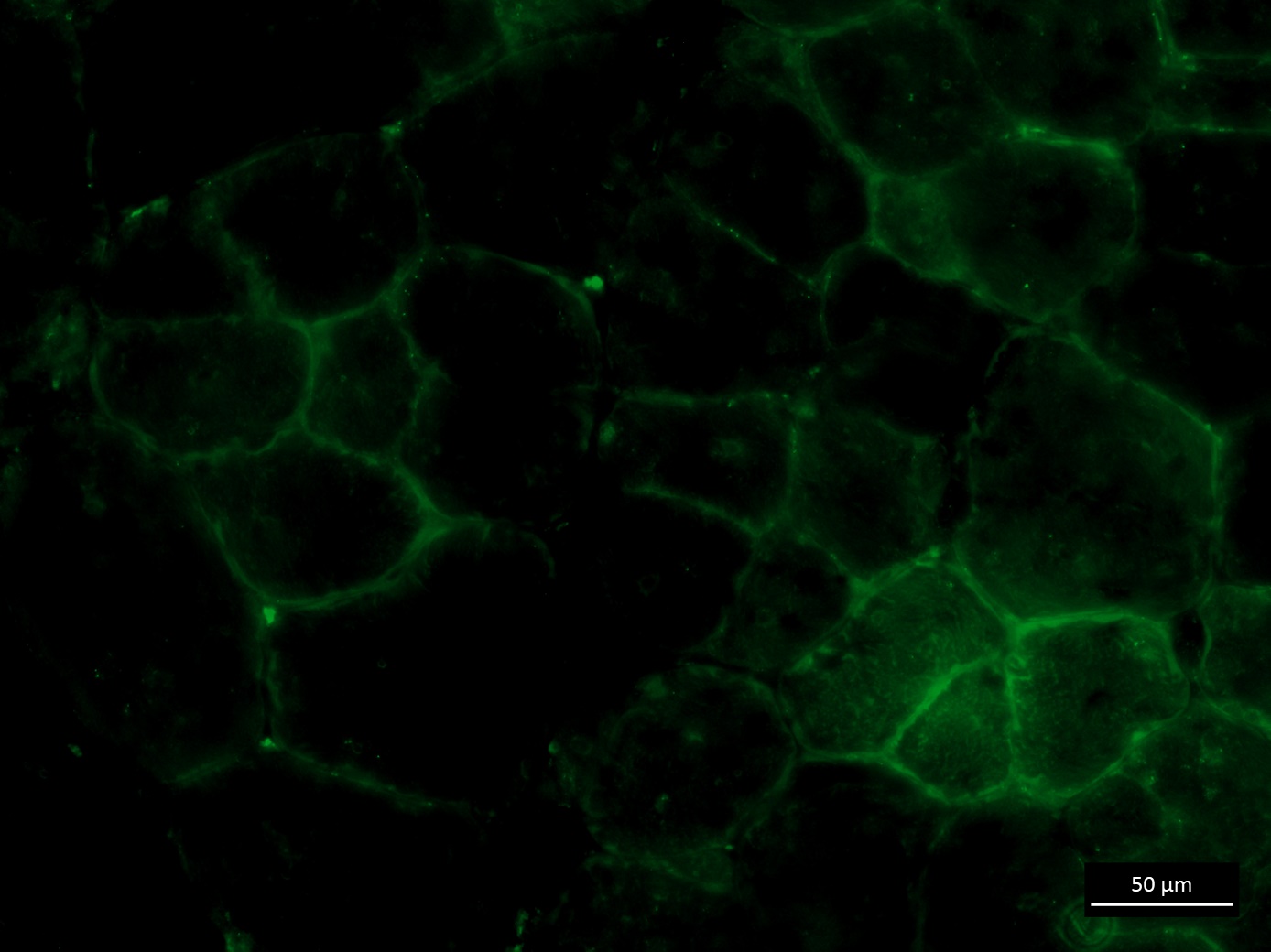

Supplement: Supplementary file 3 — Source Data Fig. 3 [file 44321_2024_31_MOESM3_ESM.zip › Figure 3/3D/51 AlphaSG.jpg]

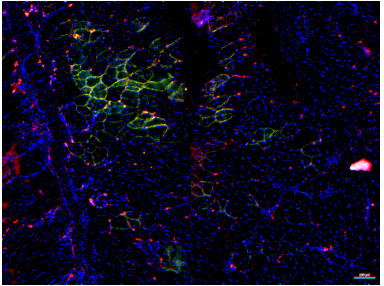

Supplement: Supplementary file 3 — Source Data Fig. 3 [file 44321_2024_31_MOESM3_ESM.zip › Figure 3/3D/51 Dapi:nNOS:AlphaSG Tile.jpg]

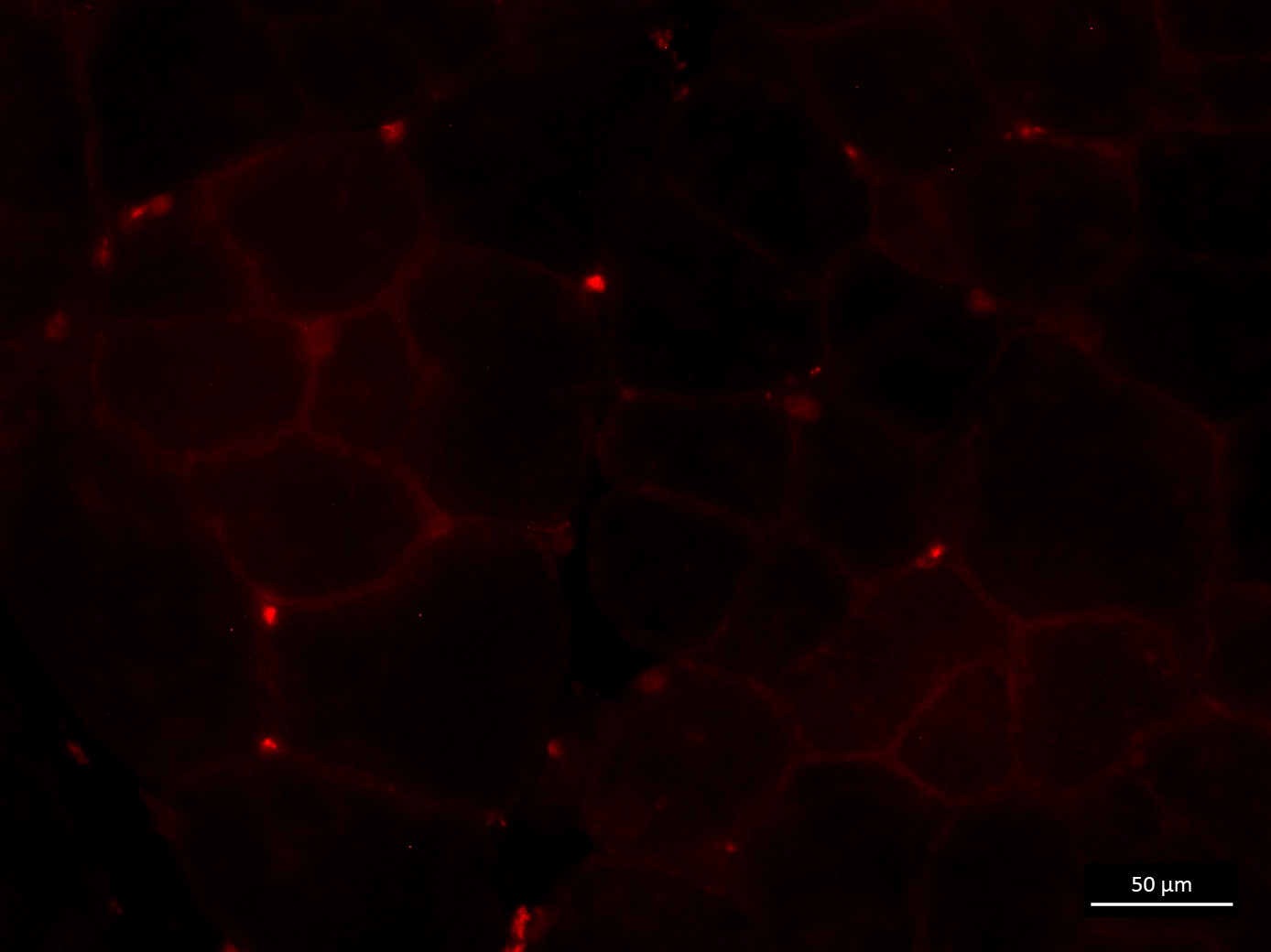

Supplement: Supplementary file 3 — Source Data Fig. 3 [file 44321_2024_31_MOESM3_ESM.zip › Figure 3/3D/51 nNOS.jpg]

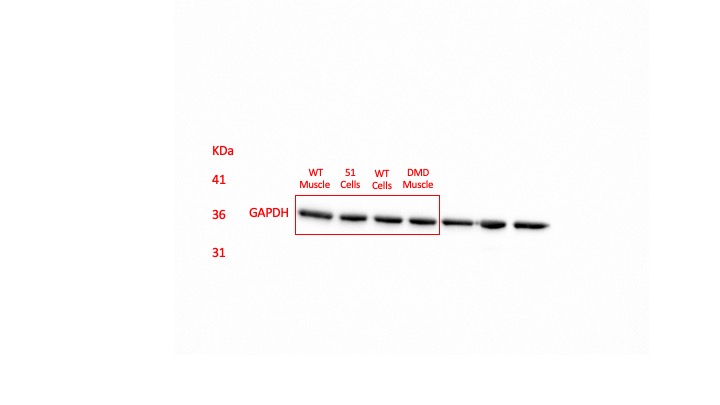

Supplement: Supplementary file 3 — Source Data Fig. 3 [file 44321_2024_31_MOESM3_ESM.zip › Figure 3/3A/GAPDH WB.jpg]

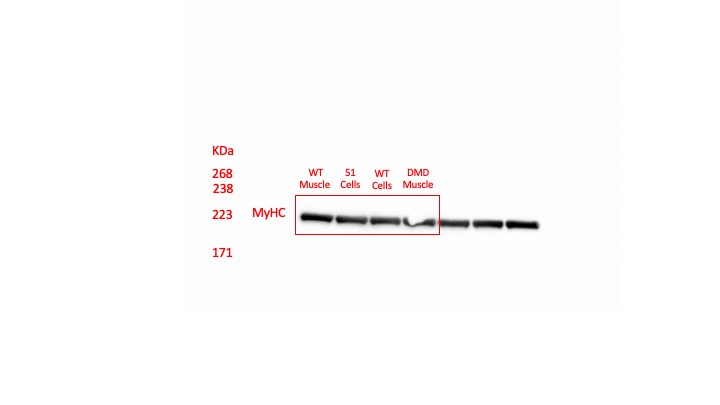

Supplement: Supplementary file 3 — Source Data Fig. 3 [file 44321_2024_31_MOESM3_ESM.zip › Figure 3/3A/MyHC WB.jpg]

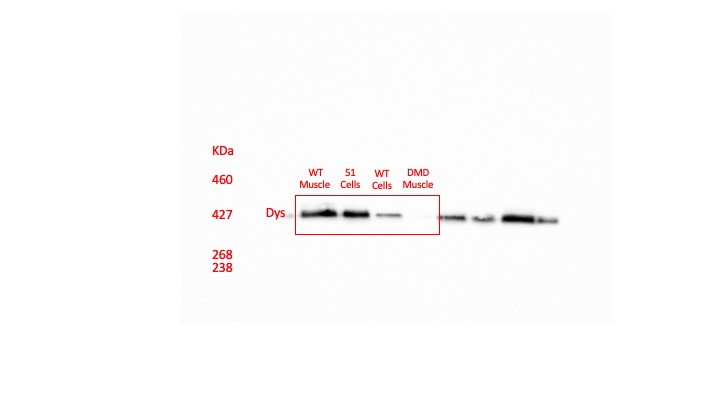

Supplement: Supplementary file 3 — Source Data Fig. 3 [file 44321_2024_31_MOESM3_ESM.zip › Figure 3/3A/Dys WB.jpg]

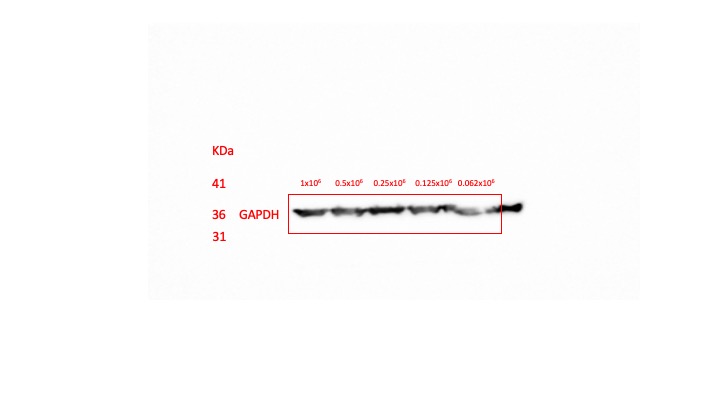

Supplement: Supplementary file 4 — Source Data Fig. 4 [file 44321_2024_31_MOESM4_ESM.zip › Figure 4/4B/GAPDH WB.jpg]

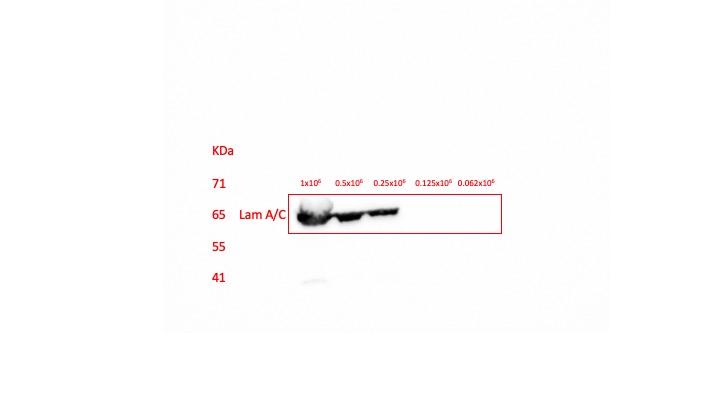

Supplement: Supplementary file 4 — Source Data Fig. 4 [file 44321_2024_31_MOESM4_ESM.zip › Figure 4/4B/Lam AC WB.jpg]

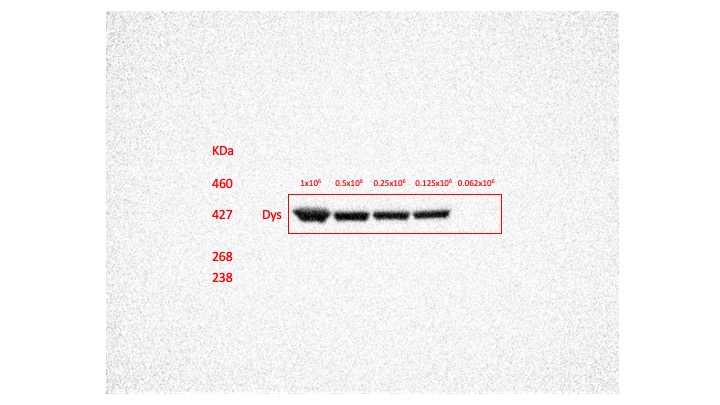

Supplement: Supplementary file 4 — Source Data Fig. 4 [file 44321_2024_31_MOESM4_ESM.zip › Figure 4/4B/Dys WB.jpg]

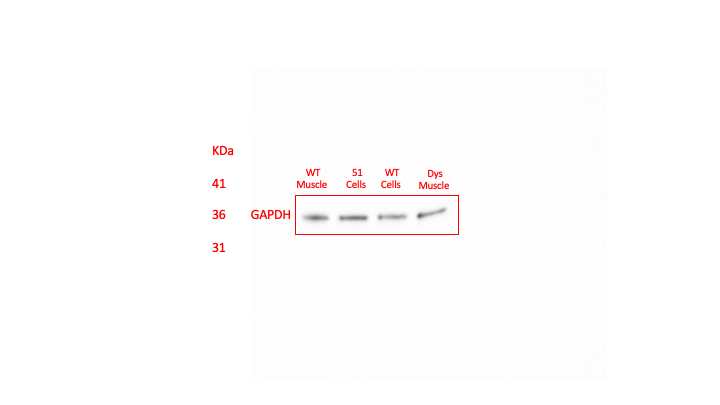

Supplement: Supplementary file 4 — Source Data Fig. 4 [file 44321_2024_31_MOESM4_ESM.zip › Figure 4/4D/GAPDH WB.tiff]

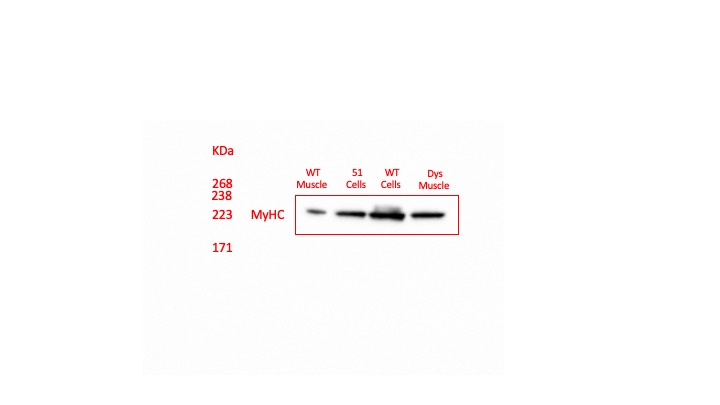

Supplement: Supplementary file 4 — Source Data Fig. 4 [file 44321_2024_31_MOESM4_ESM.zip › Figure 4/4D/MyHC WB.jpg]

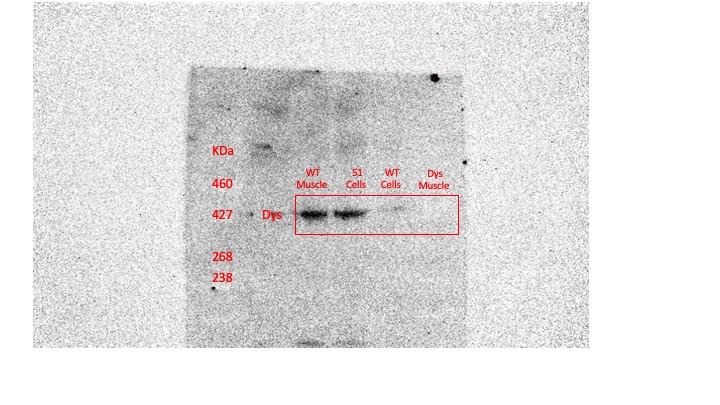

Supplement: Supplementary file 4 — Source Data Fig. 4 [file 44321_2024_31_MOESM4_ESM.zip › Figure 4/4D/Dys WB.jpg]

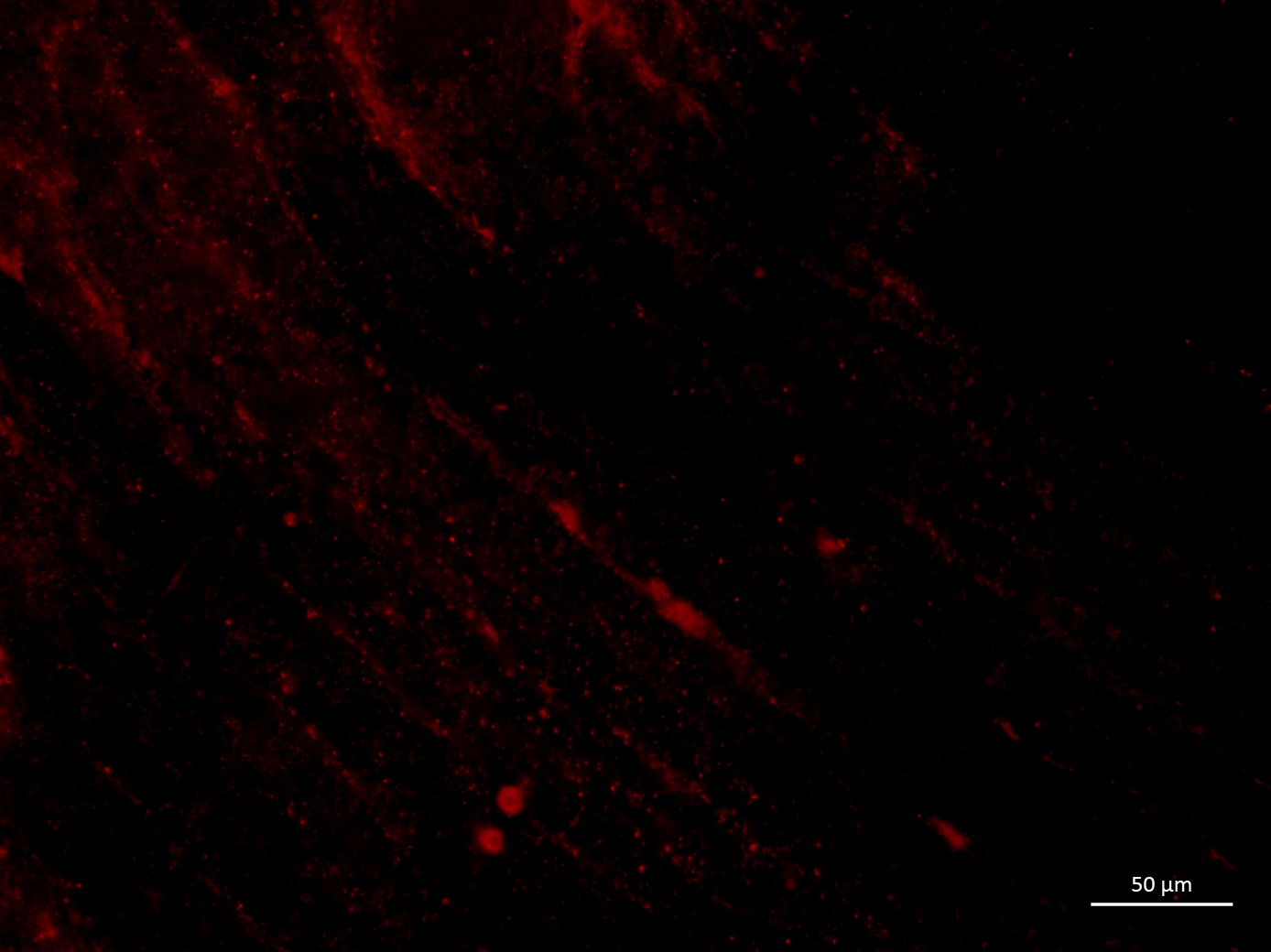

Supplement: Supplementary file 4 — Source Data Fig. 4 [file 44321_2024_31_MOESM4_ESM.zip › Figure 4/4A/snRNA In situ in vivo.jpg]

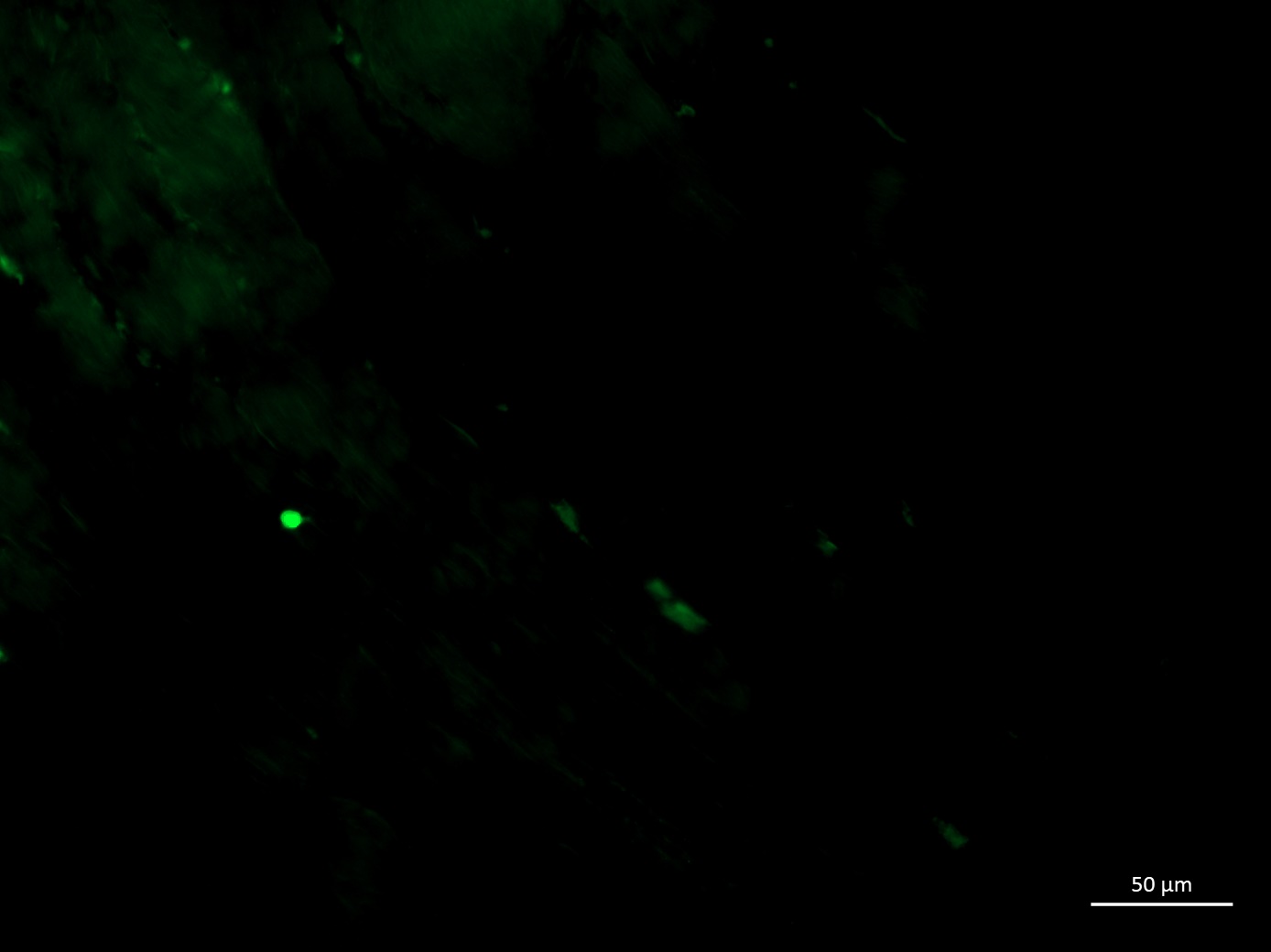

Supplement: Supplementary file 4 — Source Data Fig. 4 [file 44321_2024_31_MOESM4_ESM.zip › Figure 4/4A/LAM AC In situ in vivo.jpg]

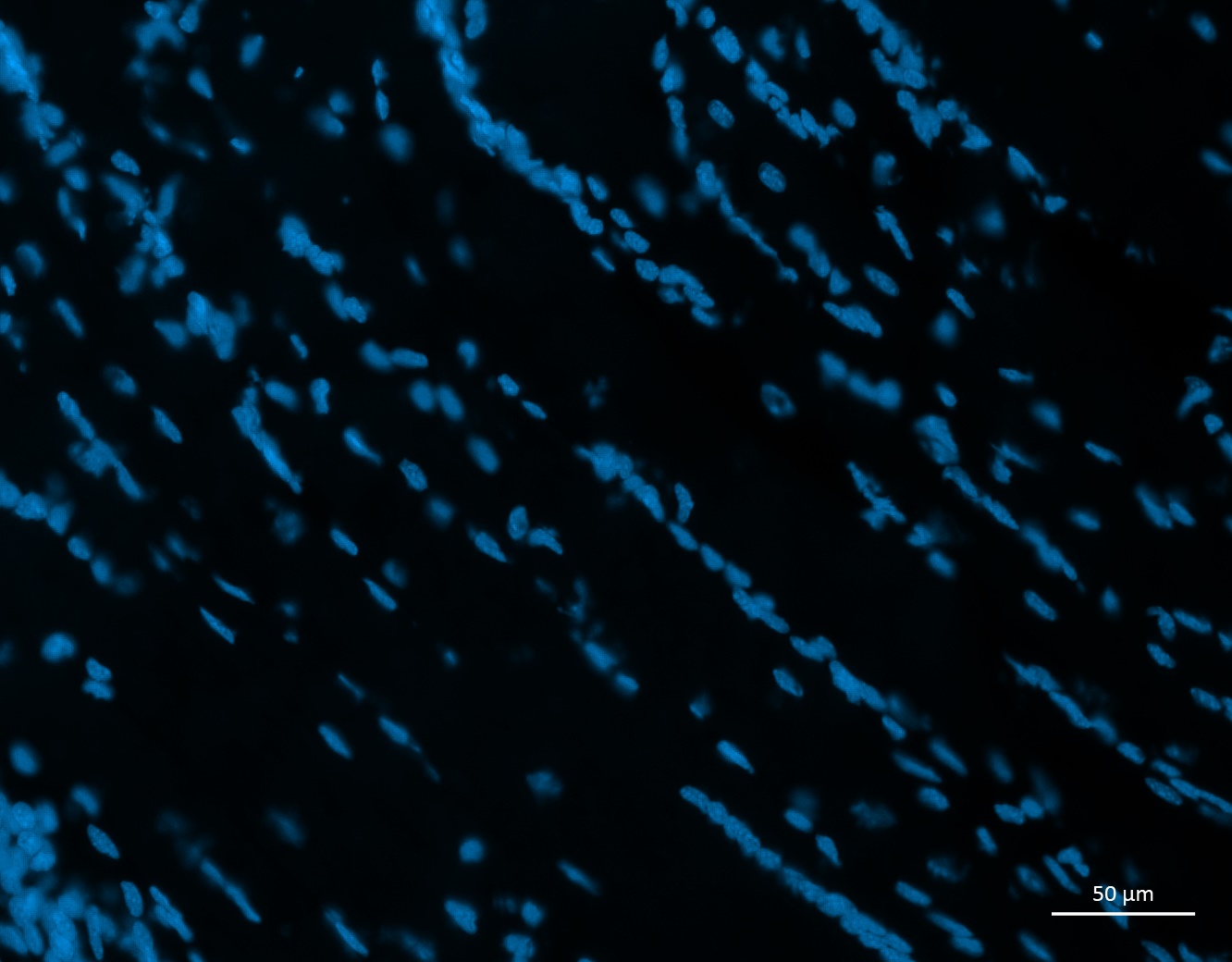

Supplement: Supplementary file 4 — Source Data Fig. 4 [file 44321_2024_31_MOESM4_ESM.zip › Figure 4/4A/DAPI In situ in vivo.jpg]

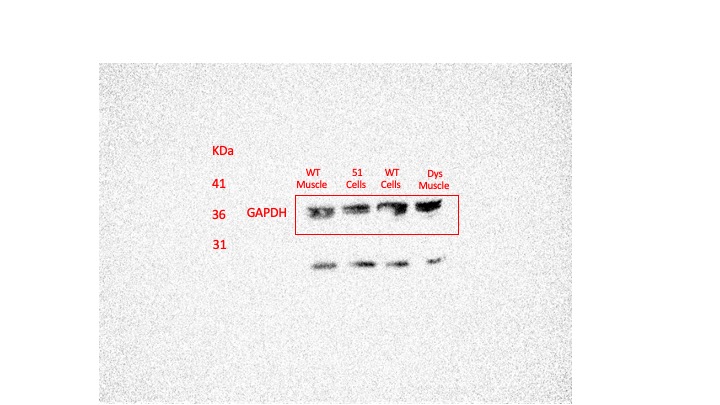

Supplement: Supplementary file 5 — Source Data Fig. 5 [file 44321_2024_31_MOESM5_ESM.zip › Figure 5/5A/GAPDH WB.jpg]

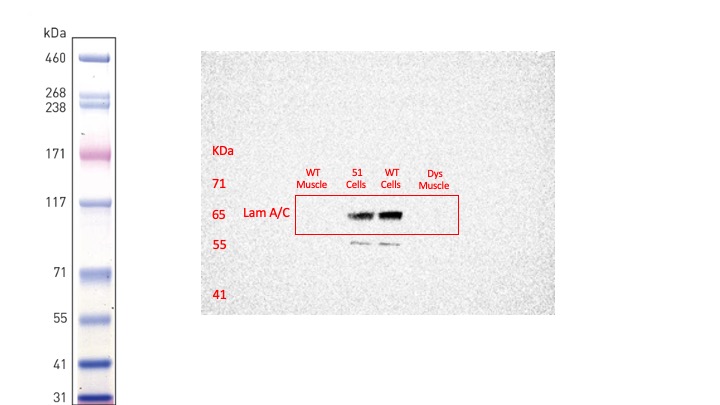

Supplement: Supplementary file 5 — Source Data Fig. 5 [file 44321_2024_31_MOESM5_ESM.zip › Figure 5/5A/Lam AC WB.jpg]

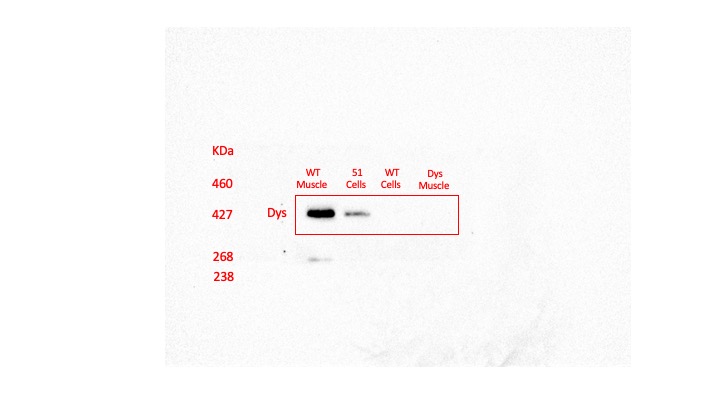

Supplement: Supplementary file 5 — Source Data Fig. 5 [file 44321_2024_31_MOESM5_ESM.zip › Figure 5/5A/Dys WB.jpg]

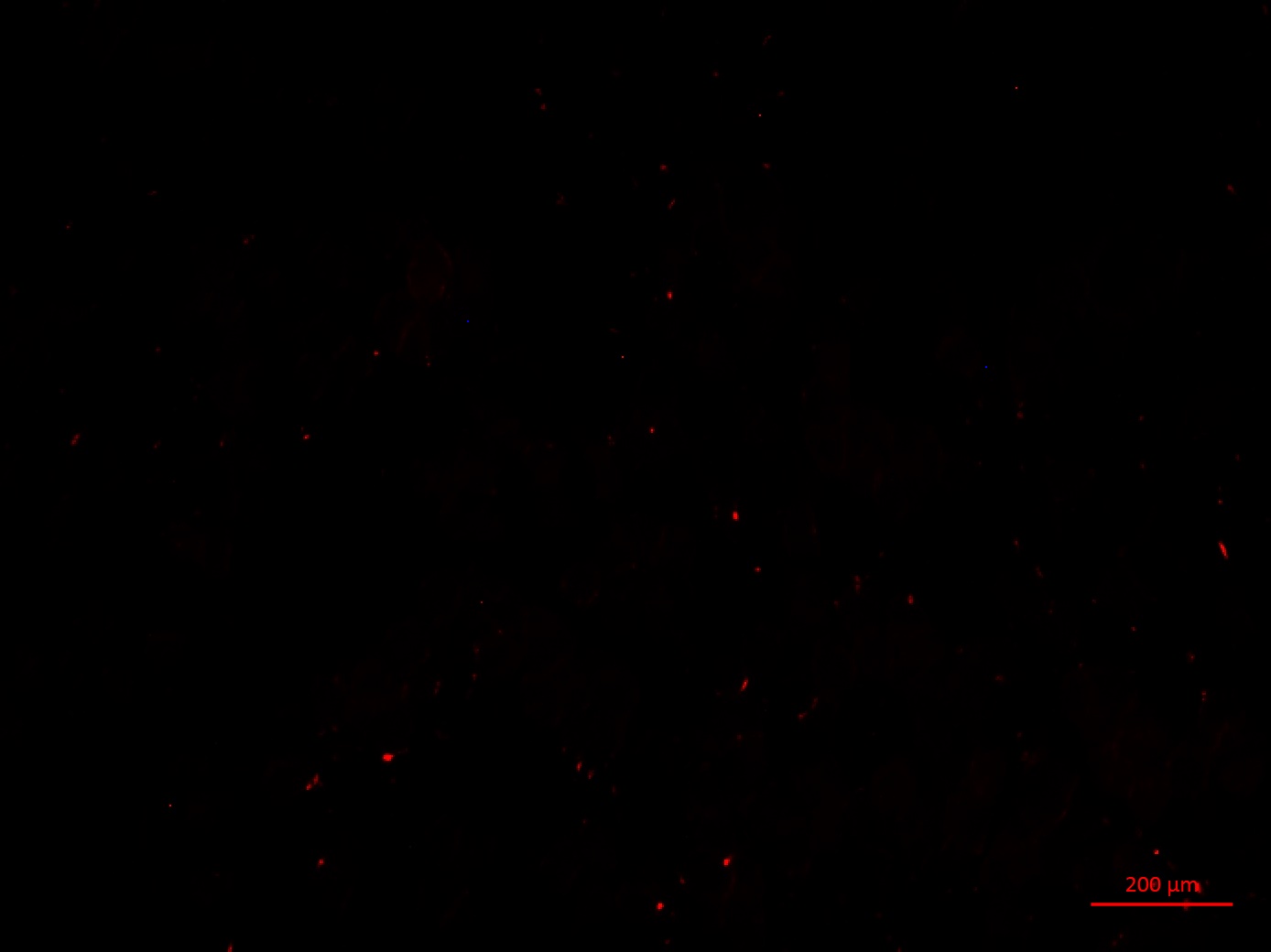

Supplement: Supplementary file 5 — Source Data Fig. 5 [file 44321_2024_31_MOESM5_ESM.zip › Figure 5/5C/LAM AC 51.jpg]

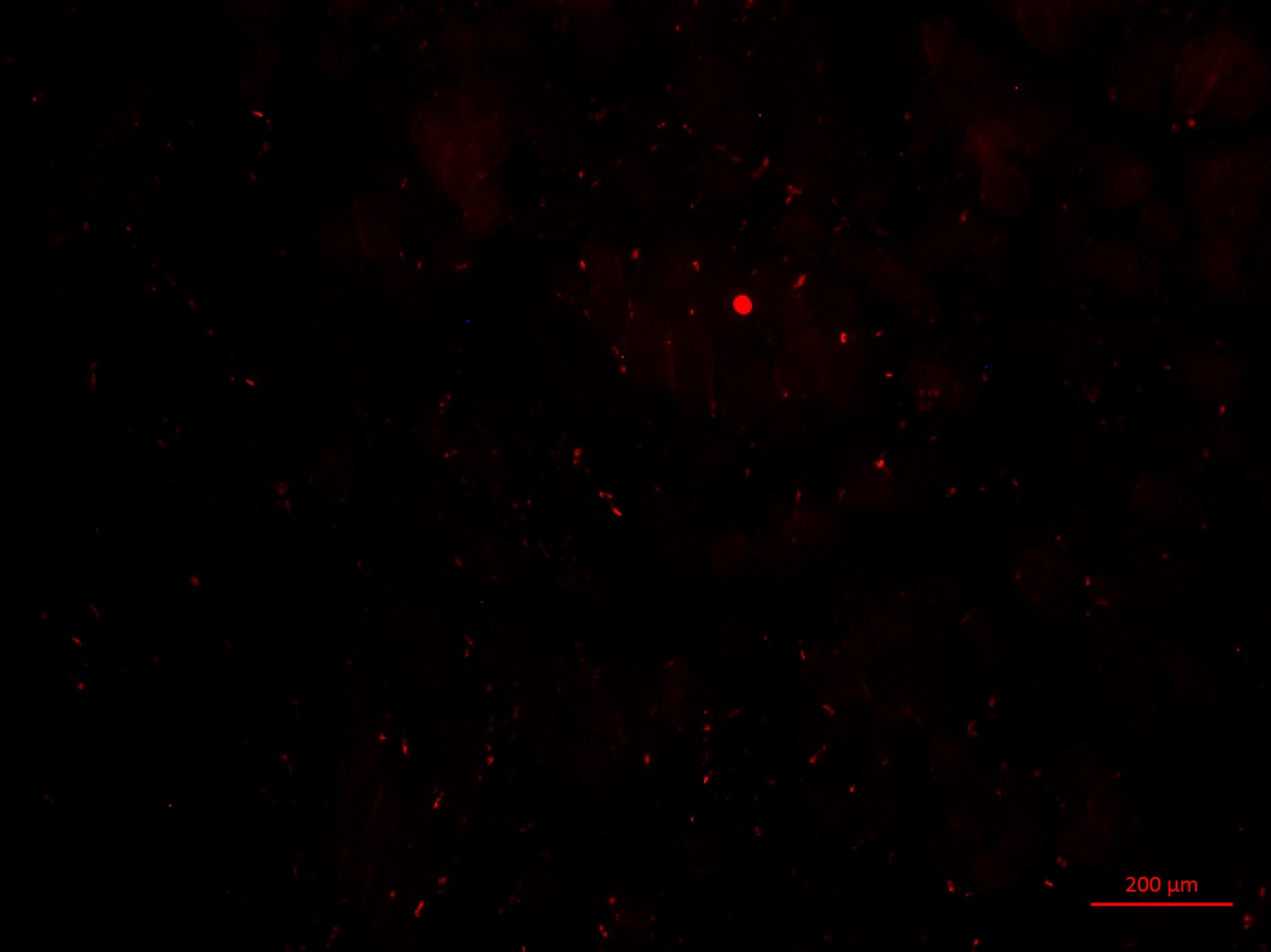

Supplement: Supplementary file 5 — Source Data Fig. 5 [file 44321_2024_31_MOESM5_ESM.zip › Figure 5/5C/LAM AC WT.jpg]

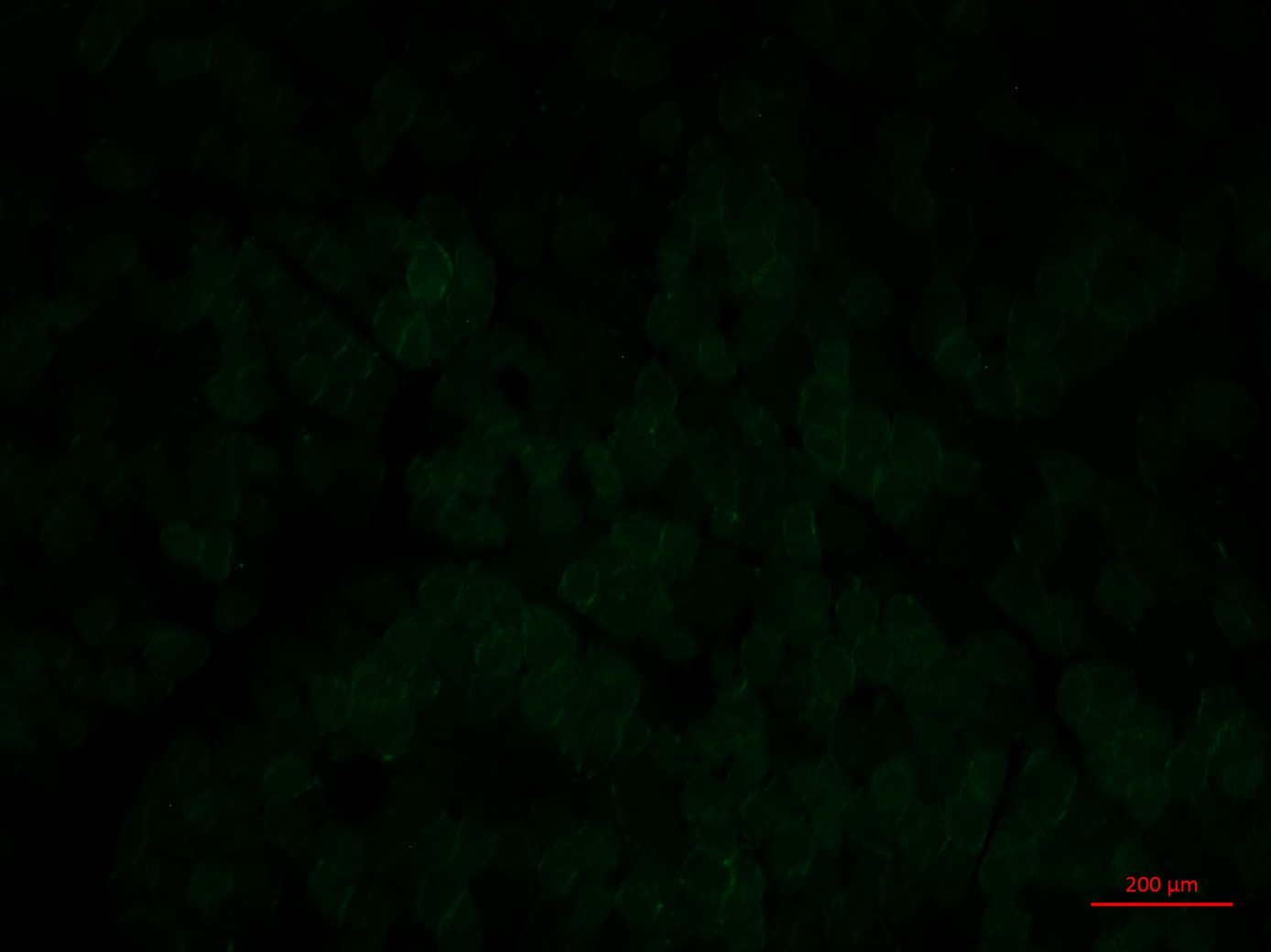

Supplement: Supplementary file 5 — Source Data Fig. 5 [file 44321_2024_31_MOESM5_ESM.zip › Figure 5/5C/DYS 51.jpg]

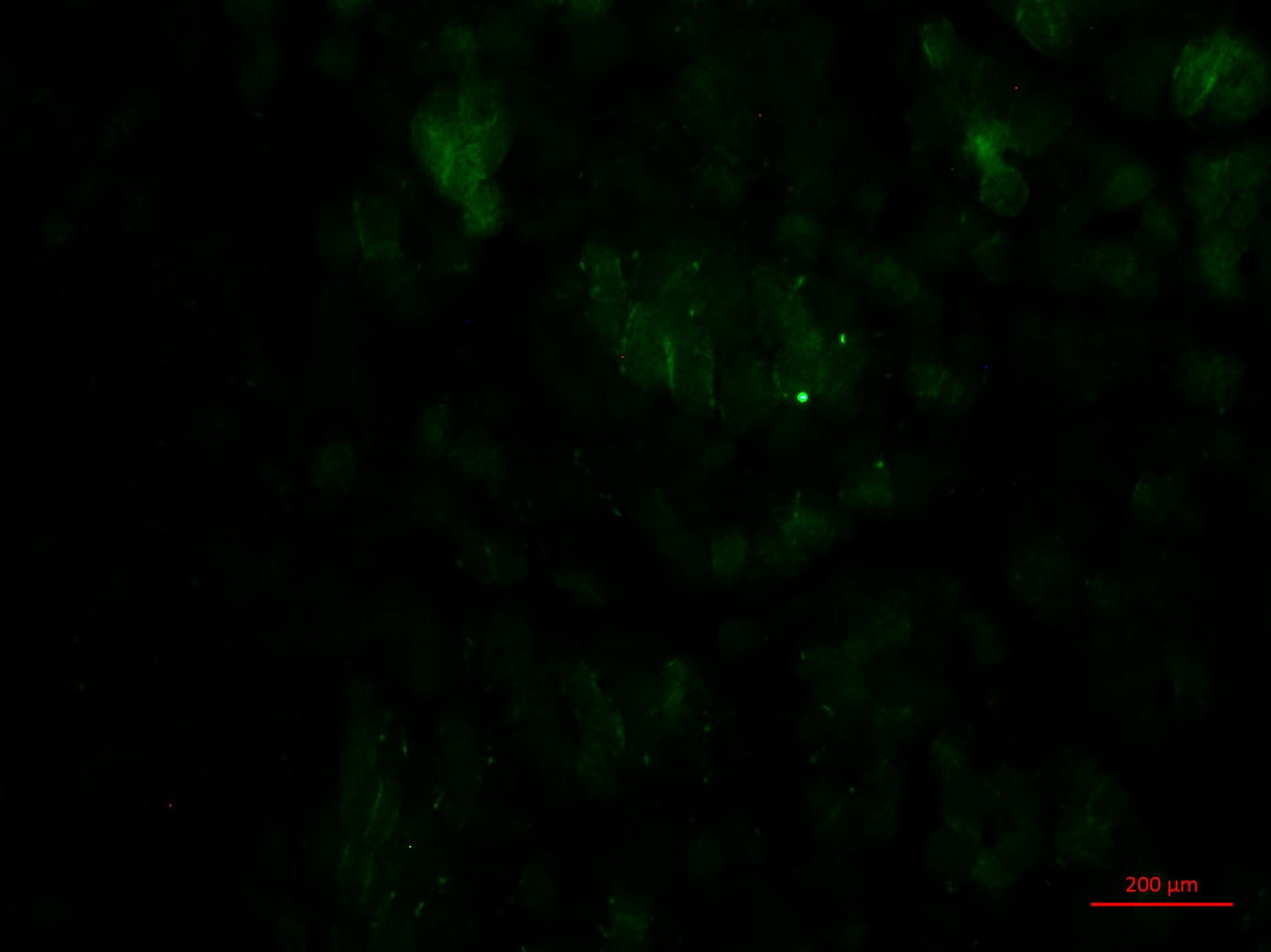

Supplement: Supplementary file 5 — Source Data Fig. 5 [file 44321_2024_31_MOESM5_ESM.zip › Figure 5/5C/DYS WT.jpg]

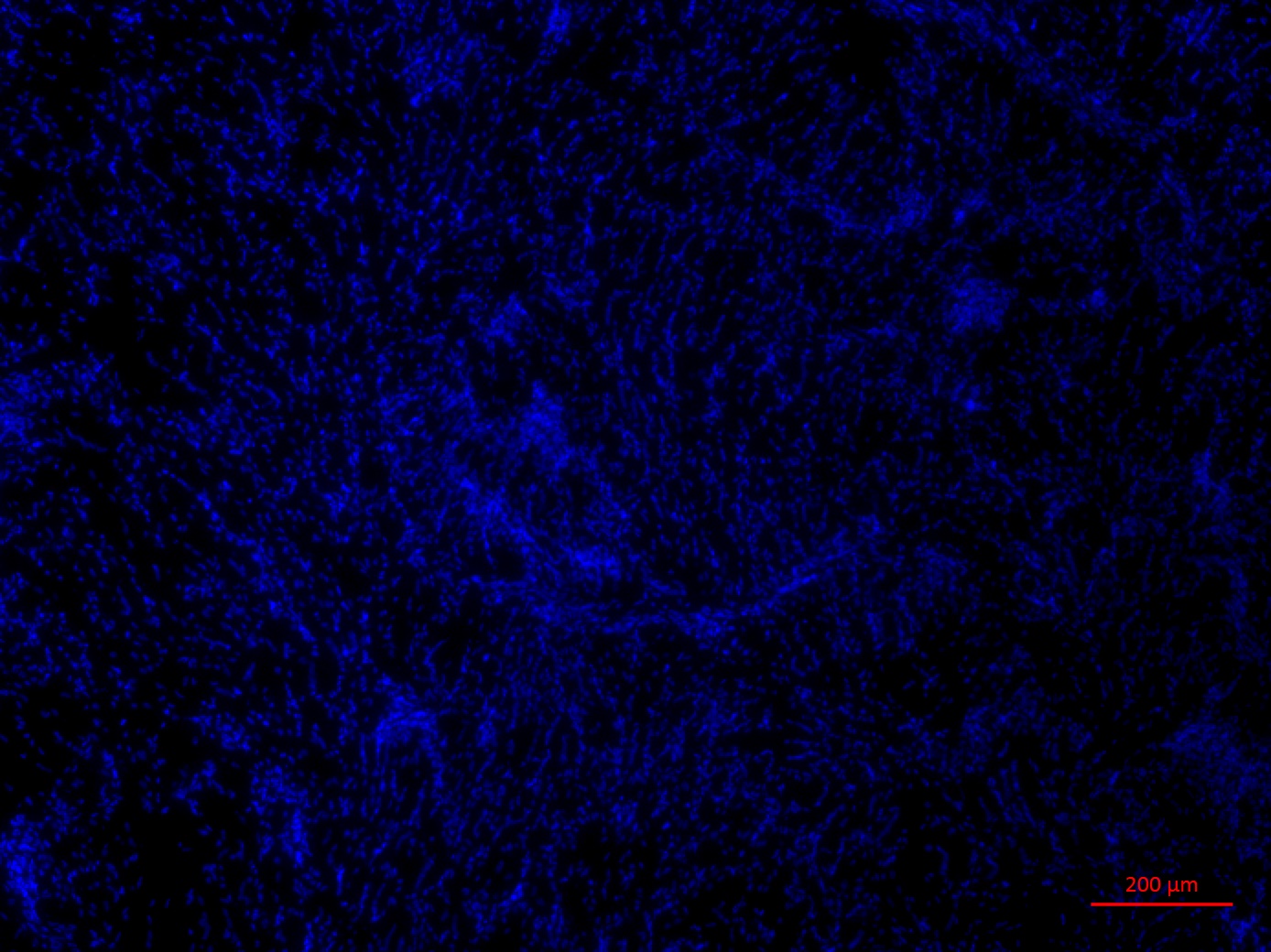

Supplement: Supplementary file 5 — Source Data Fig. 5 [file 44321_2024_31_MOESM5_ESM.zip › Figure 5/5B/DAPI WT.jpg]

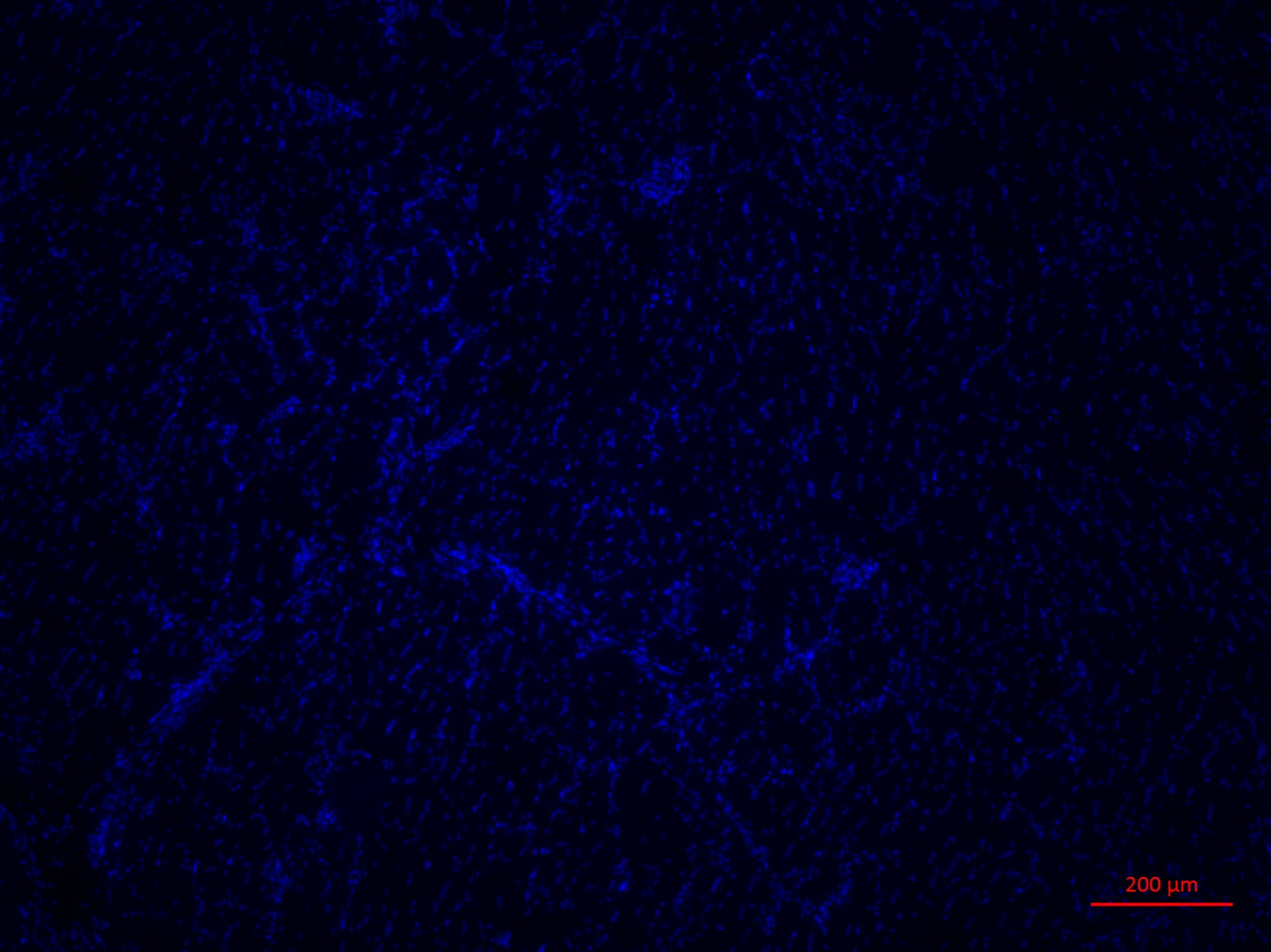

Supplement: Supplementary file 5 — Source Data Fig. 5 [file 44321_2024_31_MOESM5_ESM.zip › Figure 5/5B/DAPI 51.jpg]
