# Supplementary material for: Cell-mediated exon skipping normalizes dystrophin expression and muscle function in a new mouse model of Duchenne Muscular Dystrophy
Source: EMBO Mol Med. 2024 Mar 4;16(4):19. doi: 10.1038/s44321-024-00031-3 (PMC11018779; doi:10.1038/s44321-024-00031-3)
Supplement: Supplementary file 8 — Expanded View Figures [file 44321_2024_31_MOESM8_ESM.pdf]

Expanded View Figures

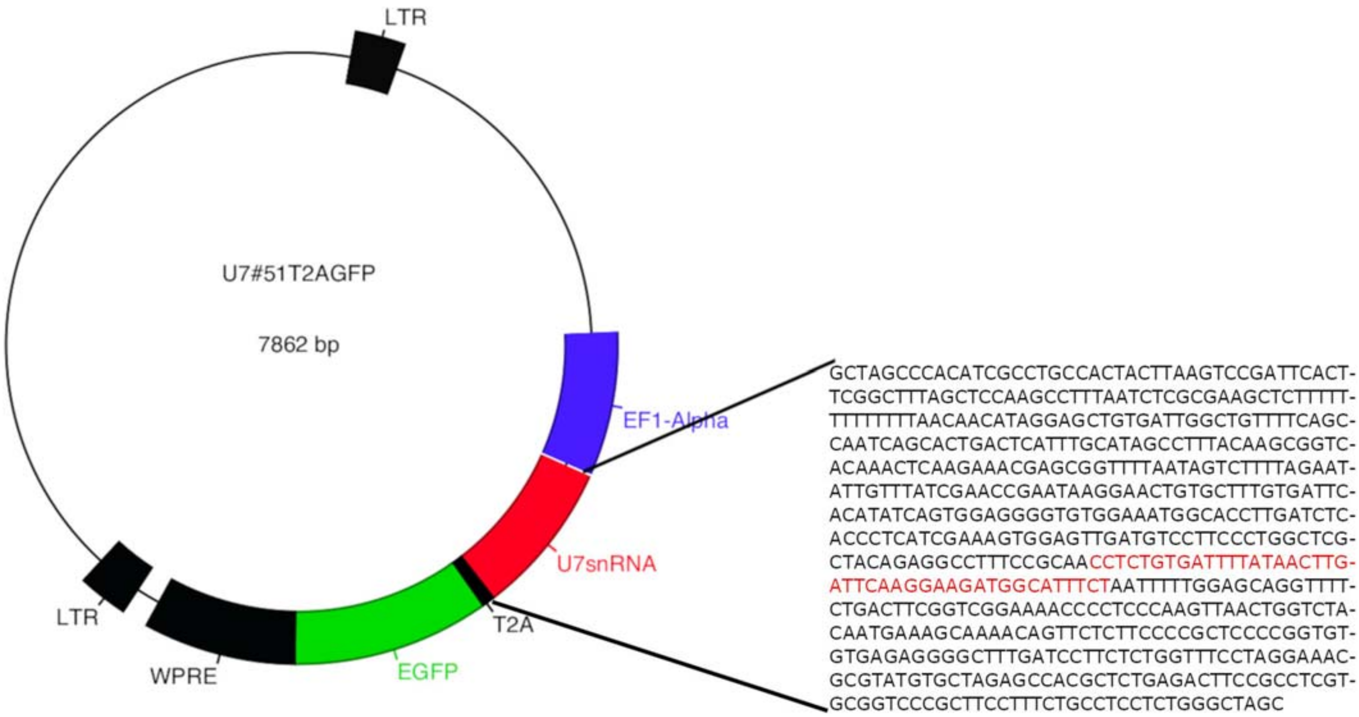

Figure EV1. Map of l lentiviral vector U7#51T2AGFP and sequence of the snRNA.

Schematic overview of the lentivector U7#51T2AGFP derived from pCDH.EFK.MCH.T2A.GFP, and sequence of the U7 snRNA with underlined in red the antisense sequence. Source data are available online for this figure

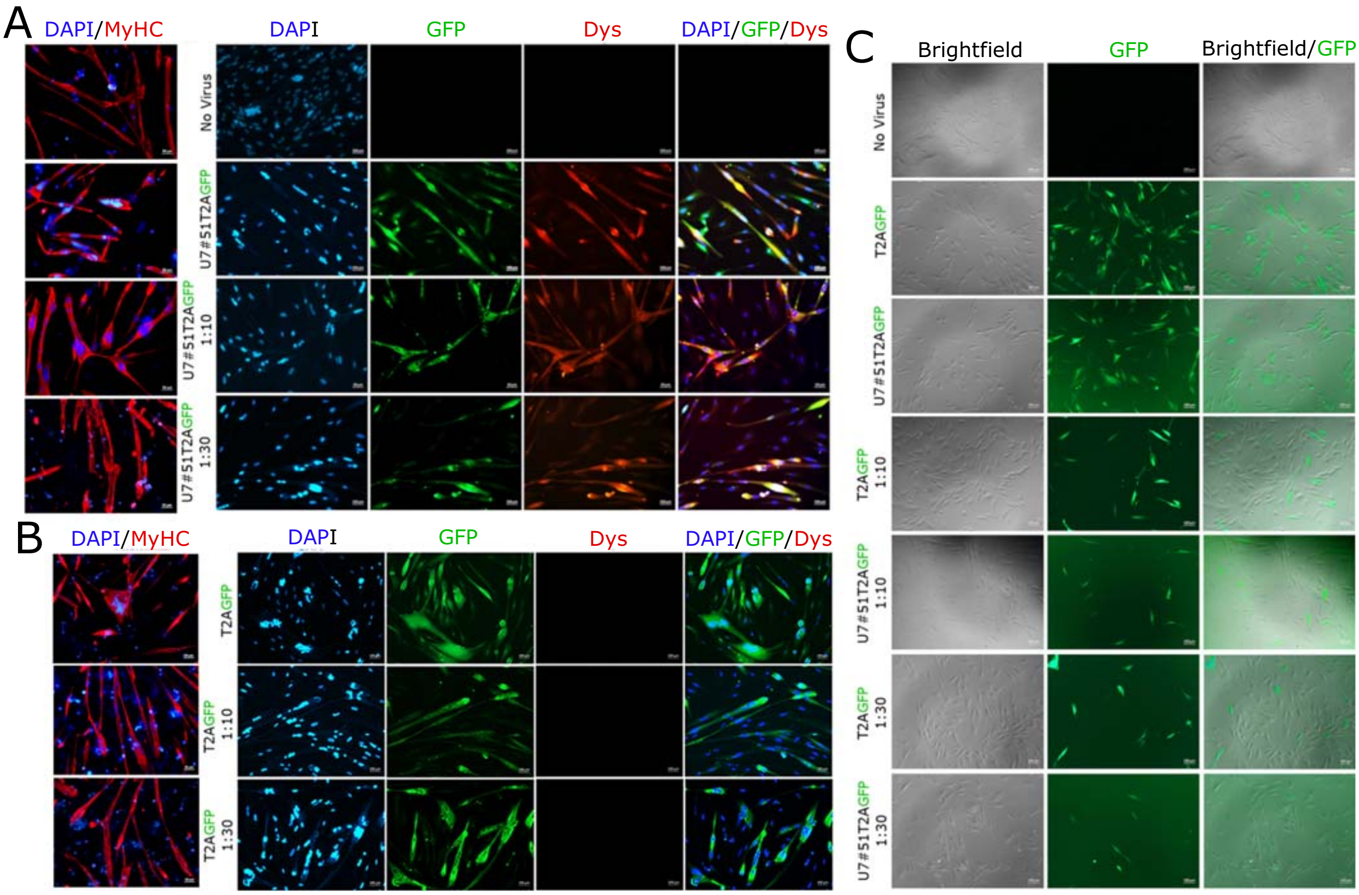

Figure EV2. Cell-mediated exon skipping induced by lentiviral vector U7#51T2AGFP.

(**A**) Representative IF images showing expression of Dystrophin and GFP in a DMD-U7/DMD TIM cells co-culture ($n = 3$ biological replicates). (**B**) Representative IF images showing expression of Dystrophin and GFP in a DMD-GFP/DMD TIM cells co-culture ($n = 3$ biological replicates). (**C**) Representative live analysis showing expression of GFP in a DMD-U7 or DMD-GFP/DMD TIM cells co-culture ($n = 3$ biological replicates). Data Information: Experiments have been replicated for at least three times. Source data are available online for this figure

© The Author(s)

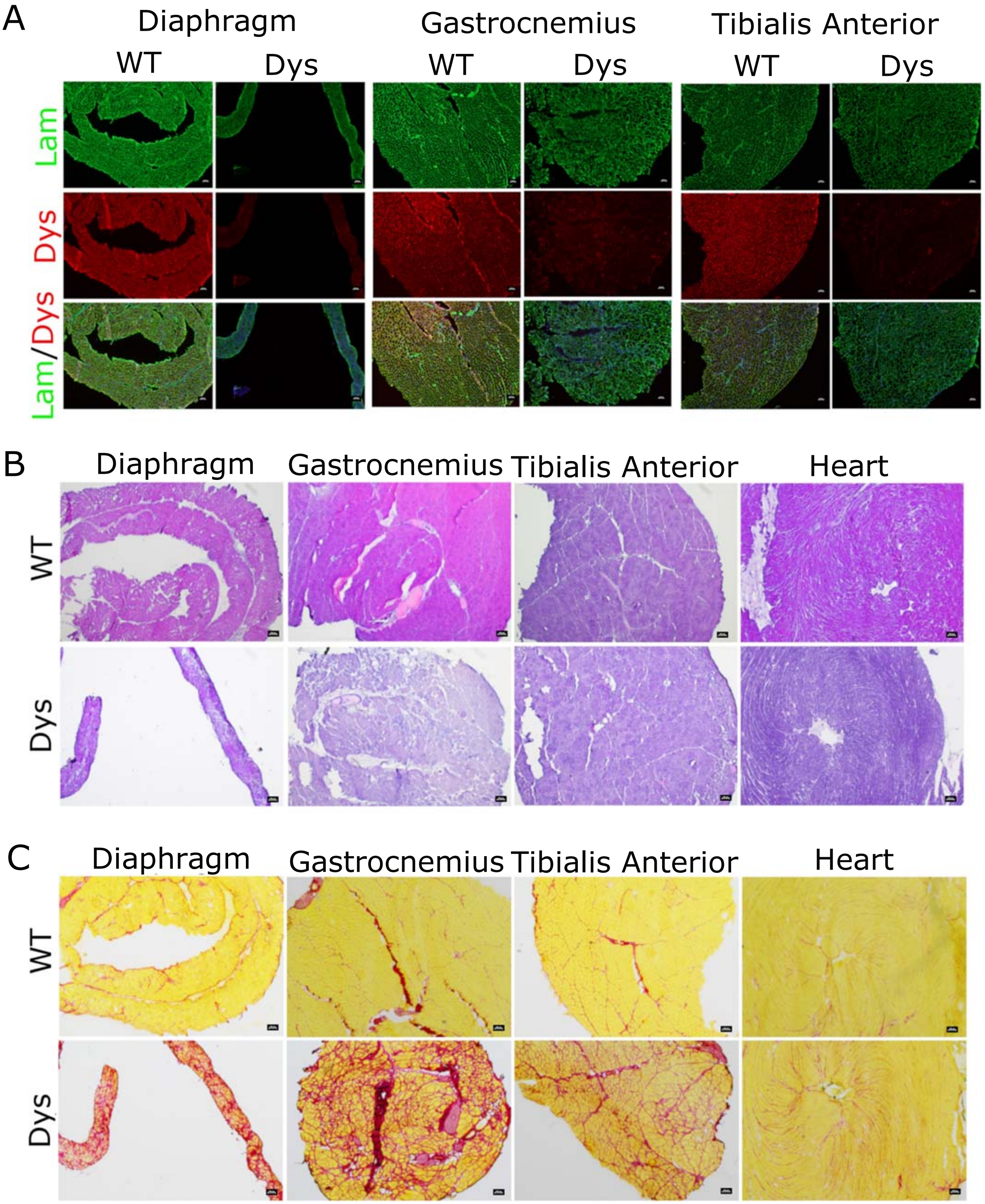
A
Diaphragm
Gastrocnemius
Tibialis Anterior
WT
Dys
WT
Dys
WT
Dys
Lam
Dys
Lam/Dys
B
Diaphragm
Gastrocnemius
Tibialis Anterior
Heart
WT
Dys
C
Diaphragm
Gastrocnemius
Tibialis Anterior
Heart
WT
Dys

Figure EV3. Phenotypical characterization of NSG-mdx-D51 mice.

Phenotypical characterization of 6 months old NSG-mdx-D51 mice by (**A**). Representative IF images showing expression of dystrophin and Laminin of Diaphragm; Gastrocnemius; Tibialis Anterior ($n = 6$ biological replicates). (**B**) Representative Haematoxylin and Eosin staining of Diaphragm; Gastrocnemius; Tibialis Anterior; Heart. ($n = 6$ biological replicates). (**C**) Representative Sirus Red staining of Diaphragm; Gastrocnemius; Tibialis Anterior; Heart ($n = 6$ biological replicates). Data Information: Experiments have been replicated for at least three times. Source data are available online for this figure

 © The Author(s)

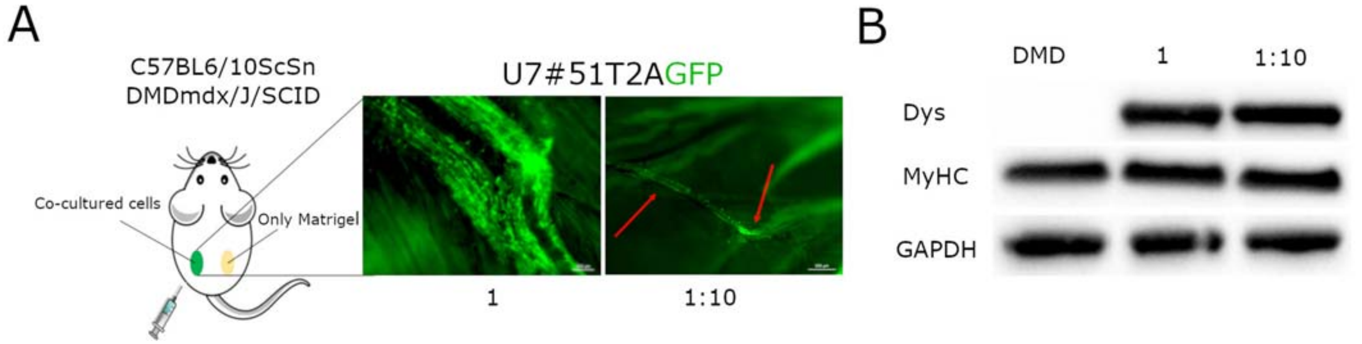

Figure EV4. Transplantation of DMD-U7 cells in a Matrigel plug under the back skin of mdx/SCID mouse.

(**A**) Representative IF images showing expression of GFP in a DMD-U7/DMD TIM cells co-culture in a matrigel plug implanted under the dorsal skin of mdx/SCID mice ($n = 3$ biological replicates). (**B**) Representative WB image of Dys expression from matrigel plug implanted under the dorsal skin of mdx/SCID. Samples are from left to right: DMD: DMD TIM cells; 1: DMD U7 cells; 1:10: DMD U7 cells co-cultured with a 10-fold excess of DMD TIM cells. ($n = 3$ biological replicates). Data Information: Experiments have been replicated for at least three times. Source data are available online for this figure

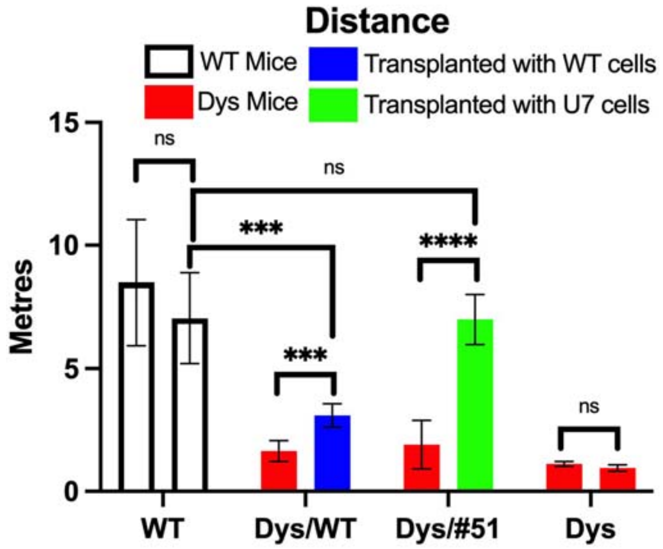

Figure EV5. Improving of mice motility after cells transplant.

Treadmill motility assay analysis before and after transplantation with either 5×10^5 WT TIM or 5×10^5 DMD-U7 cells ($n = 6$ biological replicates). Data Information: Data are shown as mean ± SD. *$P < 0.05$, **$P < 0.01$, ***$P < 0.001$, ****$P < 0.0001$. Multiple *t*-test are followed by Bonferroni correction unpaired *t*-test. The mean differences between two group were calculated with 2-way anova. Experiments have been replicated for at least three times. Source data are available online for this figure

 © The Author(s)
